# Computerized assessments of emotional expression and emotional reactivity predict negative symptoms in individuals at clinical high-risk for psychosis

**DOI:** 10.1017/S0033291726104826

**Published:** 2026-06-10

**Authors:** Claire Emilie Bertrand, Victor J. Pokorny, James M. Gold, James A. Waltz, Jason Schiffman, Lauren M. Ellman, Gregory P. Strauss, Elaine F. Walker, Scott W. Woods, Albert R. Powers, Joshua Kenney, Philip R. Corlett, Steven M. Silverstein, Vijay A. Mittal

**Affiliations:** 1 Department of Psychology, Northwestern University, Evanston, IL, USA; 2 Maryland Psychiatric Research Center, Department of Psychiatry, University of Maryland School of Medicine, Baltimore, MD, USA; 3 Department of Psychological Science, University of California, Irvine, Irvine, CA, USA; 4 Department of Psychology, Temple University, Philadelphia, PA, USA; 5 Department of Psychology, University of Georgia, Athens, GA, USA; 6 Department of Psychology, Emory University, Atlanta, GA, USA; 7 Department of Psychiatry, Yale University, New Haven, CT, USA; 8 Departments of Psychiatry, Neuroscience, and Ophthalmology, University of Rochester Medical Center, Rochester, NY, USA

**Keywords:** clinical high-risk, Psychosis, Automated facial expression, Emotional reactivity, negative symptoms, anhedonia

## Abstract

**Background:**

Negative symptoms are a core feature of psychosis and a strong predictor of functional outcome, yet they remain difficult to assess due to conceptual and methodological challenges. Although abnormalities in emotional expressivity and emotional reactivity are documented in individuals at clinical high-risk (CHR) for psychosis, these domains are typically examined independently, and their relationship remains unclear.

**Methods:**

Facial expressions were quantified using automated facial analysis (FaceReader) during clinical interviews in 101 CHR individuals and 41 healthy controls (HCs). Emotional reactivity was assessed using the International Affective Picture System (IAPS). Principal component analyses were conducted on facial expression and emotional reactivity variables within the CHR group. Associations with negative symptom domains, positive symptoms, and social functioning were examined using correlational and two-step regression analyses.

**Results:**

CHR participants showed greater disgust expression than HCs (g = 0.40, uncorrected *p* = .0025, FDR-corrected *p* = .023). Facial expression and emotional reactivity components showed minimal associations (*p* > .20). Reduced high-arousal facial expressions were associated with greater emotional expressivity deficits (*r* = −.22, *p* = .027), whereas greater happy facial expression was associated with more motivation and pleasure impairment (*r* = .21, *p* = .044). Happy facial expression explained additional variance in motivation symptoms beyond emotional reactivity (Δ*R* ^2^ = .089, *p* = .008).

**Conclusions:**

Automated facial expression captured variance in some negative symptom domains that was largely independent of emotional reactivity. These findings support the use of multimodal, objective assessments to improve characterization of negative symptoms in psychosis risk.

## Introduction

Negative symptoms are a central feature of schizophrenia and are among the strongest predictors of poor clinical and functional outcomes, often having a greater impact on long-term functioning than positive symptoms (Blanchard et al., 1998; Piskulic et al., 2012; Rabinowitz et al., 2012; Strauss et al., 2010). Despite their clinical importance, negative symptoms remain difficult to assess and treat due to longstanding conceptual and methodological challenges (Carpenter & Buchanan, 2017; Galderisi et al., 2013). Although once conceptualized as a single construct, negative symptoms are now understood to include at least two separable domains: experiential (e.g. anhedonia, avolition, asociality) and expressive symptoms (e.g. blunted affect, alogia) (Kring et al., 2013). These domains can be further differentiated into five distinct components, each showing unique relationships with symptoms and functioning (Ahmed et al., 2022; Strauss et al., 2019).

Diminished emotional expression, commonly referred to as blunted affect, is a core negative symptom of schizophrenia, defined by marked reductions in observable emotional expression across facial, vocal, and gestural behavior (Kirkpatrick et al., 2006). It is observed in approximately 30% of individuals with schizophrenia and is among the most persistent negative symptoms, strongly associated with poor social and functional outcomes (Bobes et al., 2010; Galderisi et al., 2013; Karakuş et al., 2022). This is particularly relevant in individuals at clinical high-risk (CHR) for psychosis, as diminished emotional expression has been linked to increased risk for transition to psychosis (Gupta et al., 2019; Mason et al., 2004). However, progress in understanding and treating emotional expression has been constrained by limitations in assessment. Traditional approaches rely on clinician-rated interviews, which are time-intensive, costly, subjective, and may miss subtle expressive abnormalities (Cowan et al., 2024; Strauss et al., 2020). Self-report measures are also limited, as individuals may not accurately report their own expressive behavior (Kilian et al., 2015). In contrast, advances in automated facial analysis have enabled objective, scalable, and fine-grained assessment of facial expressions (Cohen et al., 2020; Hall et al., 2024; Martin et al., 2024). Studies using these methods suggest that expressive abnormalities in CHR individuals may be emotion specific rather than global, with reduced positive (e.g. happy), but not negative (e.g. angry, sad, scared), facial expressions (Cowan et al., 2022; Gupta et al., 2019).

A related but distinct literature has focused on emotional reactivity. Meta-analytic work indicates that individuals with schizophrenia and those at CHR show altered emotional reactivity compared to healthy controls (HC), characterized by reduced positive emotion and increased negative emotion in response to pleasant stimuli, as well as greater positive emotion in response to unpleasant stimuli (Riehle et al., 2024). These alterations are present prior to illness onset and have been linked to increased anhedonia and depressive symptoms (Gruber et al., 2018; Strauss et al., 2018).

Studies in schizophrenia suggest a dissociation between emotional reactivity and emotional expression, such that outward emotional expression does not necessarily reflect individuals’ subjective emotional experience (Kring et al., 1993; Kring & Neale, 1996). However, it remains unclear whether this same dissociation is present in CHR individuals. Although prior studies have documented abnormalities in emotional reactivity, motivation, and emotional expressivity, these domains have largely been studied independently. As a result, it remains unclear how task-based emotional reactivity, objective facial expression, and clinician-rated negative symptom domains relate to one another in CHR. Clarifying these relationships may help determine whether expressive and experiential symptoms reflect partly distinct processes, refining the conceptualization and assessment of negative symptoms.

The present study addressed this gap by integrating automated facial expression analysis during clinical interviews with a computerized assessment of emotional reactivity using the International Affective Picture System (IAPS; Lang et al., 1997). Here, emotional expressivity refers to clinician-rated outward emotional expression, facial expression refers to automated measures derived from FaceReader, and emotional reactivity refers to task-based emotional responses measured using the IAPS. First, we examined group differences in facial expressions, predicting that CHR individuals would show reduced positive facial expressions and similar or increased negative expressions compared to HCs (Cowan et al., 2022; Gupta et al., 2019). Second, within the CHR group, we examined associations between facial expression components and emotional reactivity components, hypothesizing weak associations consistent with the dissociation between emotional experience and expression in schizophrenia (Kring et al., 1993; Kring & Neale, 1996). Third, we examined whether facial expression components were differentially associated with negative symptom domains and social functioning, with stronger associations expected for emotional expressivity than for motivational or experiential negative symptoms (Cohen et al., 2013; Cowan et al., 2022). Finally, we tested whether facial expression components explained variance in negative symptoms beyond emotional reactivity to evaluate the incremental clinical utility of automated facial assessment.

## Methods

### Participants

A total of 142 participants (101 CHR, 41 HC) with usable video data were recruited as part of the Clinical Assessment of Psychosis Risk (CAPR) multisite study across six sites: Northwestern University, Yale University, University of Georgia, Temple University, Emory University, and the University of California, Irvine. Participants were recruited through advertisements, campus postings, public transportation ads, and community outreach. Informed consent was obtained from all adult participants. For minors, participant assent and written consent were provided by their parents or guardians, in accordance with protocols approved by the Northwestern University Institutional Review Board (for more details on Methods, see Mittal et al., 2021).

Participants in the CHR group met criteria for progressive or persistent psychosis-risk syndromes, as determined through the Structured Interview for Psychosis-Risk Syndromes (SIPS; Miller et al., 2003; Woods et al., 2010). The Structured Clinical Interview for DSM-5 Disorders (SCID-5) was administered to assess current psychiatric diagnoses and comorbid conditions (First, 2015).

Exclusion criteria included a history of traumatic brain injury, intellectual disability or neurological disorder, any past or current psychotic disorder, and any ongoing substance use disorder other than alcohol or cannabis use. HCs were also excluded if they had any first-degree relatives with a psychotic disorder, current or past major psychopathology determined by the SCID, or any prior or current use of antipsychotic or other psychotropic medications.

### Clinical measures

The SIPS (Miller et al., 2003) is a semi-structured clinical interview to assess psychosis-risk symptoms across four domains: positive (e.g. unusual thought content, perceptual abnormalities), negative (e.g. avolition, diminished emotional expression), disorganized (e.g. disorganized communication and behavior), and general symptoms (e.g. sleep disturbance, motor abnormalities). Items are rated on a 6-point scale based on symptom severity, progression, and frequency, with domain scores calculated by summing individual item ratings. The SIPS total positive symptom score was used to assess for positive symptoms.

Negative symptoms were assessed using the Negative Symptom Inventory-Psychosis Risk (NSI-PR; Strauss et al., 2025), a semi-structured interview assessing five symptom domains: anhedonia, avolition, asociality, blunted affect, and alogia, rated from 0 (absent) to 5 (extremely severe). In addition to item-level scores, NSI-PR items are organized into two domains: motivation and pleasure (MAP) and emotional expressivity (EE), where higher scores indicate greater negative symptom severity. The MAP domain primarily reflects deficits in anticipatory pleasure, motivation, and engagement in goal-directed behavior, whereas the EE domain captures observable reductions in emotional expressivity.

Social functioning was assessed using the Global Functioning Scale: Social (GFS-S; Carrión et al., 2019). The GFS-S is rated on a 10-point scale, with higher scores indicating better current social functioning across domains, such as peer relationships, interpersonal conflicts, and social engagement.

Clinical interviews and assessments were conducted remotely via Zoom by trained staff, doctoral students, and postdoctoral professionals and were recorded with participants’ consent for data analysis.

### Emotional reactivity

Emotional reactivity was assessed using an IAPS-based picture-viewing task, in which participants rated their responses to standardized emotional stimuli. The task consisted of 90 trials (30 pleasant, 30 unpleasant, and 30 neutral images) presented in random order. After each image, participants rated their positive emotion, negative emotion, and arousal using the Self-Assessment Manikin (Strauss et al., 2017). Ratings were made on unipolar 1–5 scales, with higher scores indicating greater emotional intensity.

### Automated facial expression analysis

Facial expressions were assessed using the first 10 minutes of the SIPS clinical interview, consisting of standard demographic and background questions. This approach is consistent with prior CHR research demonstrating reliable detection of individual differences from brief, neutral interview segments (Gupta et al., 2019, 2022; Lozano-Goupil et al., 2025).

All videos were manually reviewed to ensure adequate quality, lighting, movement, background stability, and clear visibility of the face. Videos were excluded if more than 20% of frames were not analyzable by FaceReader. Following these procedures, an additional 14 participants (7 CHR, 7 HC) were excluded based on video quality. After exclusions, 94.8% of CHR data and 92.5% of HC data were retained for analysis.

Facial expressions were analyzed using FaceReader version 9.0, an automated software that uses deep learning-based convolutional neural networks to classify facial expressions (Noldus Information Technology, 2021). FaceReader quantifies seven discrete emotions (neutral, happy, sad, angry, surprised, scared, and disgusted) on a frame-by-frame basis at 25 frames per second, with values ranging from 0 (no expression) to 1 (full expression). FaceReader also provides dimensional ratings of arousal and valence, reflecting overall emotional intensity and overall level of positivity to negativity expressed on the face. FaceReader has demonstrated convergence with other automated programs and human coding approaches, and has been used in prior research with CHR populations (Gupta et al., 2022; Lewinski et al., 2014). FaceReader scores were averaged across the recording to yield a single mean value per emotion (Gupta et al., 2019). Zero intensity values were retained, and frames with failed detection were excluded.

Stability was evaluated by comparing average emotion scores across different segments of the interview, with consistency quantified as correlations across participants.

### Statistical analysis

Categorical variables (sex, race, ethnicity, and medication status) were compared using chi-square tests or Fisher’s exact tests when cell counts were less than five. Continuous variables (e.g. age, education, clinical symptom scores, task measures) are reported as means and standard deviations and were compared between groups using Welch’s two-sample *t*-tests. Race categories were collapsed for statistical testing due to small subgroup sizes.

*Aim 1a: Group differences in facial expression*. Each of the nine FaceReader emotion variables was examined separately using one-way analyses of variance with group as the between-subjects factor. Follow-up robustness analyses were conducted using linear mixed-effects models that included group, sex, age, site, and antipsychotic medication use as fixed effects, with assessor included as a random intercept to account for potential rater- and site-related differences. Assessor effects were quantified using intraclass correlation coefficients (ICCs).

*Aim 1b: Group differences in emotional reactivity*. Group differences in IAPS self-report ratings were examined using Welch’s two-sample *t*-tests. Separate tests were conducted for each combination of stimulus category (pleasant, neutral, unpleasant) and rating type (positive emotion, negative emotion, arousal), resulting in nine comparisons.

*Aim 2a–2b: Principal components analysis (PCA).* PCA was conducted within the CHR group to identify latent dimensions of emotional expression and emotional reactivity. Separate PCAs were performed for FaceReader variables and IAPS ratings. The number of components was determined based on scree plot inspection, and components were rotated using oblique Promax rotation.

*Aim 3: Correlational analyses.* Within the CHR group, Pearson correlations were computed to examine associations between PCA-derived facial expression components, PCA-derived emotional reactivity components, and clinical/functional outcomes. Predictor variables included the three PCA-derived facial expression components and the three PCA-derived emotional reactivity components. Outcomes included the NSI-PR emotional expressivity (EE), NSI-PR motivation and pleasure (MAP), current social functioning (GFS-S), and total positive symptoms on the SIPS. This resulted in 24 total correlation tests. Two-tailed *p* values were obtained for each correlation, and false discovery rate (FDR) correction was applied.

*Aim 4: Incremental validity of facial expression using two-step sequential regression*. To test whether facial expressions explained variance in clinical and functional outcomes beyond emotional reactivity, three separate two-step sequential multiple regressions were conducted in the CHR group, with NSI-PR EE, NSI-PR MAP, and current social functioning as outcomes. In each model, Step 1 included sex, age, and the PCA-derived emotional reactivity component showing the strongest prior association with the outcome. Step 2 added the PCA-derived facial emotion component showing the strongest prior association with that same outcome. Sex and age were included as covariates given their relationship with clinical presentation (Maric et al., 2003). This approach was used to test whether automated facial expression measured during the clinical interview explained unique variance beyond task-based emotional reactivity, given the established use of IAPS-based paradigms for assessing emotional reactivity and the potential utility of automated facial expression as a scalable complementary measure. Model fit was assessed using adjusted *R* ^2^ and ΔR^2^. Sensitivity analyses used linear mixed-effects models with sex, age, and emotional reactivity as fixed effects and assessor included as a random intercept. Likelihood ratio tests evaluated the incremental contribution of facial emotion components. FDR correction was applied across outcomes. An overview of the analytic pipeline is provided in Supplementary Figure S1.

## Results

### Demographics and clinical characteristics

A total of 142 participants (101 CHR and 41 HC) were included in the final analyses. Demographic characteristics are presented in Table 1. CHR and HC participants did not differ significantly in age, sex, years of education, ethnicity, or race. CHR participants were more likely to report psychiatric medication use and to meet criteria for current SCID diagnoses compared to HCs (Fisher’s *p* < .001). For clinical symptom measures, CHR participants showed significantly more positive symptoms compared to HC participants, *t*(139.72) = −21.62, *p* < .001, as well as significantly lower social functioning, *t*(110.73) = 6.95, *p* < .001.

**Table 1.** Demographic, clinical, and task characteristics of healthy control (HC) and clinical high-risk (CHR) groups Table 1. long description.

	HC (*N* = 41)	CHR (*N* = 101)	Statistics
Age (Years)			*t*(71.65) = −1.08, *p* = .29
Mean (SD)	22.56 (4.02)	23.35 (3.79)	
Sex			χ^2^ (1) = 0.68, *p* = .41
Male, n (%)	14 (34.1%)	42 (41.6%)	
Female, n (%)	27 (65.9%)	59 (58.4%)	
Education (Years)			t(62.90) = 0.89, *p* = .38
Mean (SD)	14.59 (2.30)	14.22 (1.84)	
Missing, n (%)	0	5 (4.9%)	
Ethnicity			χ^2^ (1) = 0.00, *p* = .98
Hispanic, n (%)	4 (9.8%)	10 (9.9%)	
Not Hispanic, n (%)	37 (90.0%)	91 (90.1%)	
Race, n (%)			χ^2^(1) = 0.002, *p* = .96
Asian	16 (39.0%)	15 (14.9%)	
American Indian	0	1 (1.0%)	
Black	2 (4.9%)	14 (13.9%)	
White	22 (53.7%)	52 (51.5%)	
More than one race	1 (2.4%)	13 (12.9%)	
Missing	0	6 (5.9%)	
Medication, n (%)			Fisher’s *p* < .001
Psychiatric medication	0	34 (33.7%)	
Antidepressant	0	21 (20.8%)	
Antianxiety	0	6 (5.9%)	
Antipsychotic	0	8 (7.9%)	
Stimulant	0	15 (14.9%)	
Mood stabilizer	0	6 (5.9%)	
Missing	4 (9.8%)	9 (8.9%)	
Current diagnoses, n (%)			Fisher’s *p* < .001
Major depressive disorder	0	16 (15.8%)	
Persistent depressive disorder	0	20 (19.8%)	
Generalized anxiety disorder	0	46 (45.5%)	
Social anxiety disorder	0	49 (48.5%)	
Bipolar disorder	0	15 (14.8%)	
Post-traumatic stress disorder	0	21 (20.8%)	
Attention-deficit/hyperactivity disorder	0	34 (33.7%)	
Obsessive compulsive disorder	0	23 (22.8%)	
Substance use disorder	0	26 (25.7%)	
Clinical measures			
SIPS, total positive	1.45 (1.45)	10.40 (3.47)	*t*(139.72) = −21.62, *p* < .001
GFS-S, current social	8.59 (0.84)	7.29 (1.31)	*t*(110.73) = 6.95, *p* < .001
NSI-PR, EE	2 (1.41)	3.05 (3.82)	*t*(5.06) = −1.31, *p* = .25
NSI-PR, MAP	7 (2.83)	11.30 (7.75)	*t*(4.09) = −2.83, *p* = .046

*Note:* Clinical measures include the Structured Interview for Psychosis-Risk Syndromes (SIPS) total positive symptom score, the Negative Symptom Inventory-Psychosis Risk (NSI-PR) motivation and pleasure (MAP) and emotional expressivity (EE) domains, and the Global Functioning Scale–Social (GFS-S). Current diagnoses were assessed using the Structured Clinical Interview for DSM-5. Participants may meet criteria for more than one diagnosis. Group comparisons were conducted using Welch’s *t*-tests for continuous variables and chi-square or Fisher’s exact tests for categorical variables, as appropriate.

### Group differences in facial emotion expressions

**Figure 1.**
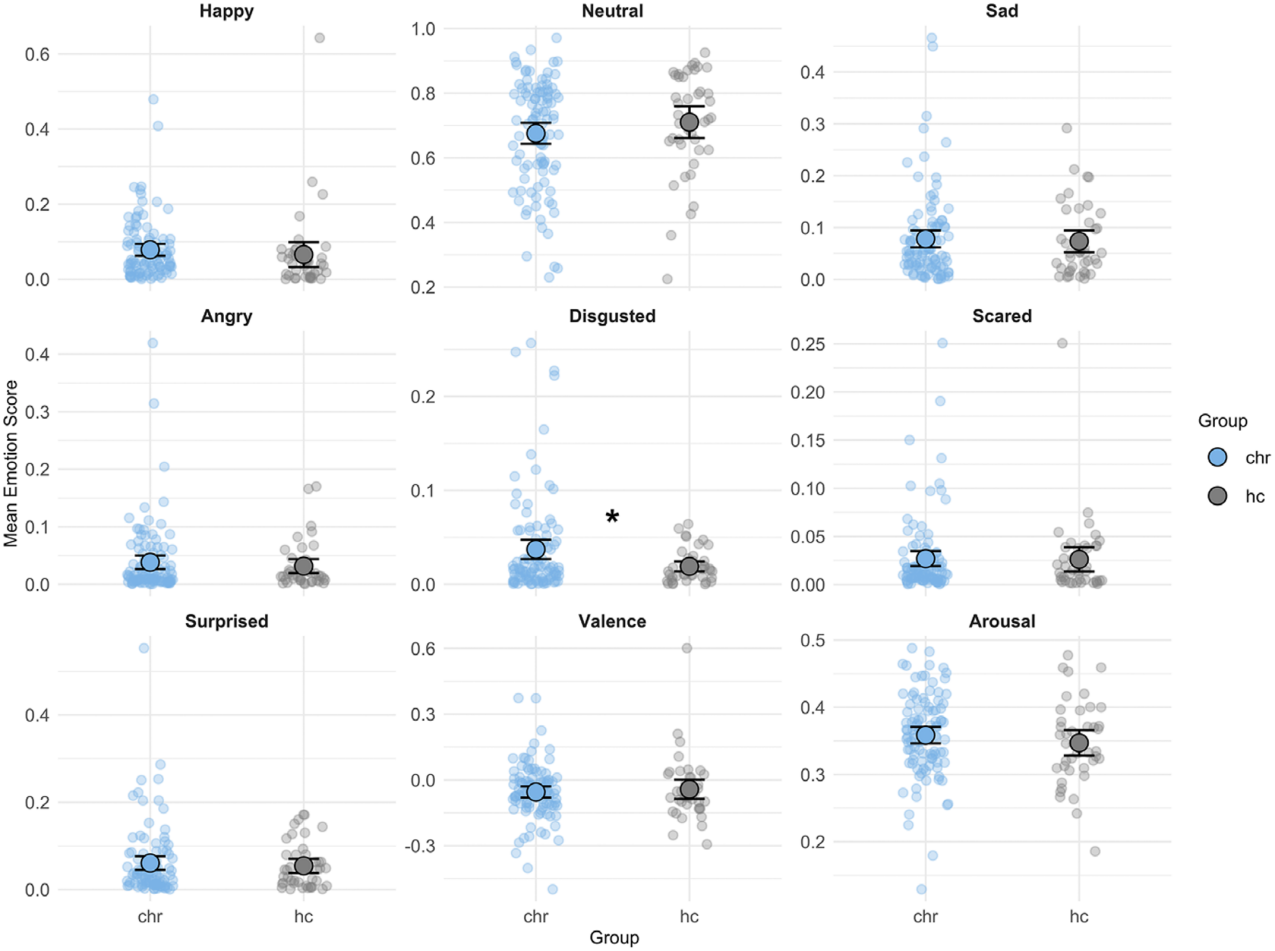
FaceReader facial emotion expressions across clinical high-risk (CHR) and healthy controls (HC) groups. A significant group difference was observed for Disgust (uncorrected *p* = .0025), which survived false discovery rate (FDR) correction (*p* = .0228). Uncorrected *p* values were Neutral (*p* = .25), Arousal (*p* = .32), Angry (*p* = .44), Happy (*p* = .50), Surprised (*p* = .58), Valence (*p* = .63), Sad (*p* = .73), and Scared (*p* = .92). Error bars represent 95% confidence intervals. Figure 1. long description.

Among the nine FaceReader variables examined, the only significant group difference was for disgust, with CHR participants showing higher mean disgust expression than HCs (see Figure 1; Hedges’ g = 0.40, 95% CI [0.03, 0.76], *p* = .0025, FDR-corrected *p* = .023). No significant group differences were observed for the remaining facial expressions. Full descriptive statistics are in Supplementary Table S1.

Follow-up linear mixed-effects models were conducted that included sex, age, and antipsychotic use as covariates, with assessor modeled as a random intercept. Results were similar in adjusted analyses, although the disgust effect did not survive FDR correction (see Supplementary Figure S2).

Assessor-related variability was quantified using ICCs derived from the mixed-effects models. ICCs indicated small-to-moderate between-assessor variability for several emotions (ICCs = 0.11–0.21 for happy, surprised, neutral, and valence), whereas assessor-related variability was negligible for disgust and arousal (see Supplementary Table S2). This suggests that the observed disgust was unlikely to be driven by assessor-related factors. Facial expression measures showed stability across segments of the interview (Spearman–Brown reliability = .97–.99).

### Group differences in emotional reactivity

**Figure 2.**
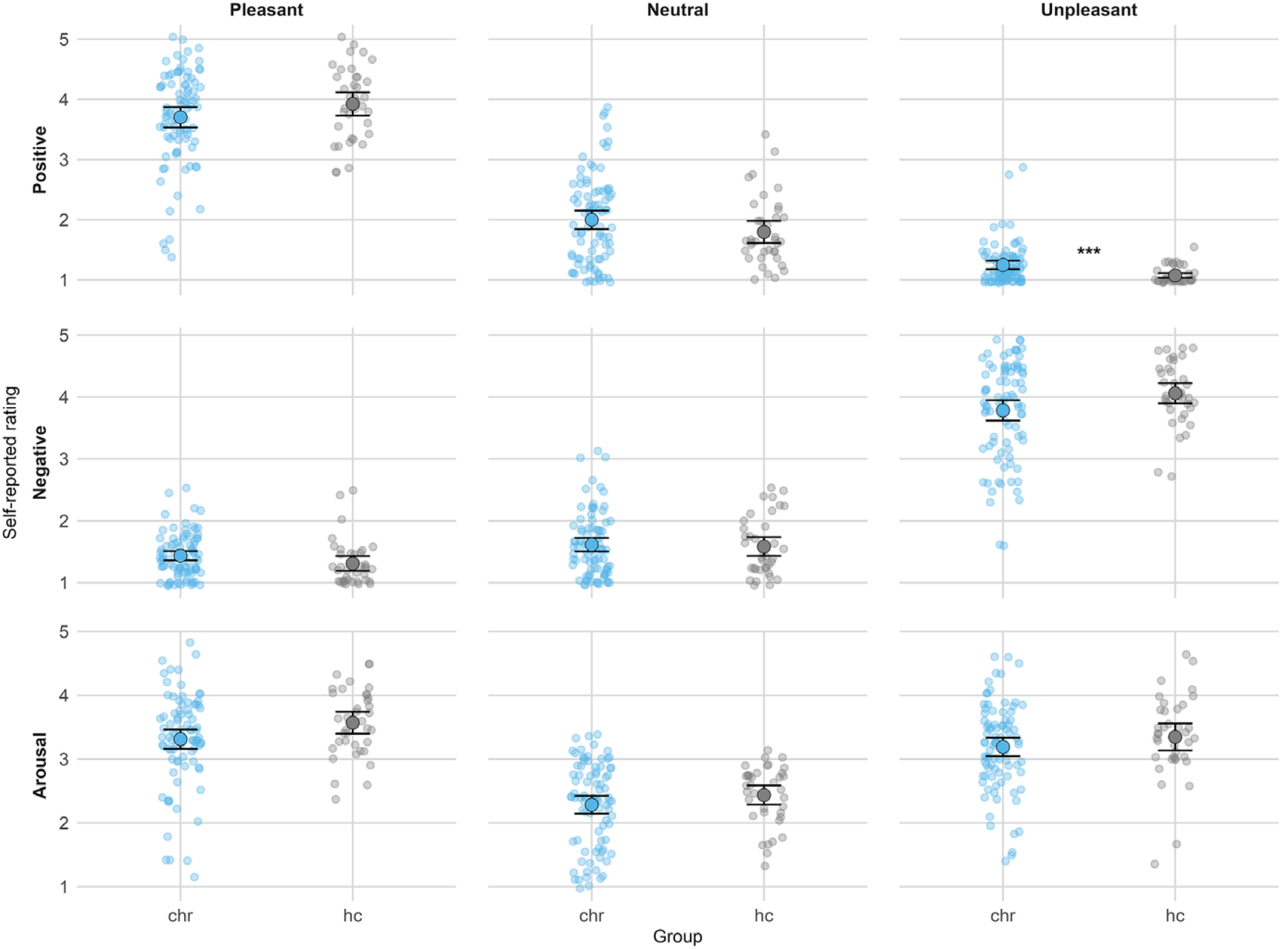
Self-reported emotional experience ratings during the International Affective Picture System (IAPS) task across clinical high-risk (CHR) and healthy controls (HC) groups. Columns are organized by stimulus category (pleasant, neutral, unpleasant), and rows are organized by rating type (positive, negative, arousal). Points represent individual participants, circles indicate group means, and error bars represent 95% confidence intervals. A significant group difference was observed for positive emotion ratings to unpleasant stimuli (uncorrected *p* < .001), which survived false discovery rate (FDR) correction (*p* < .01). Group differences were observed for positive emotion ratings to pleasant stimuli (*p* = .042), negative emotion ratings to unpleasant stimuli (*p* = .022), and arousal ratings to pleasant stimuli (*p* = .030), though these did not survive FDR correction. All remaining comparisons were non-significant (*p* ≥ .08). Figure 2. long description.

Compared to HCs, CHR participants reported significantly greater positive emotion ratings to unpleasant stimuli (see Figure 2 and Supplementary Table S3; g = 0.60, 95% CI [0.21, 0.99], *p* < .001, FDR-corrected *p* < .001). CHR participants also reported significantly lower positive emotion ratings to pleasant stimuli (g = −0.35, 95% CI [−0.69, −0.01], *p* = .042), indicating reduced emotional reactivity. Nominal group differences were additionally observed for negative emotion ratings to unpleasant stimuli (g = −0.39, 95% CI [−0.77, 0.00], *p* = .022) and arousal ratings to pleasant stimuli (g = −0.38, 95% CI [−0.77, 0.00], *p* = .030), but these effects did not survive FDR correction. Overall, these findings indicate altered emotional reactivity in CHR participants across both pleasant and unpleasant conditions.

### PCA-derived facial expression and general emotional reactivity components

PCA of FaceReader facial emotion expressions within the CHR group yielded a three-component solution (see Supplementary Figure S3), accounting for 64.1% of the variance (see Supplementary Table S4). The first component (27.6%) reflected a negative-valence facial expression dimension, with primary loadings on sadness and anger. The second component (18.4%) reflected a high-arousal facial expression dimension, with strong loadings on scared and surprised expressions. The third component (18.2%) reflected a happy facial expression dimension, with a primary loading on happy expressions.

PCA of IAPS emotional reactivity yielded a three-component solution (see Supplementary Figure S4), accounting for 68.0% of the variance (see Supplementary Table S5). The first component (29.3%) reflected general emotional reactivity, with strong loadings on congruent emotional responses. The second component (21.1%) and third component (17.6%) reflected emotional ambivalence, characterized by negative emotion ratings to pleasant stimuli and positive emotion ratings to unpleasant stimuli, respectively.

### Associations between facial expression components, emotional reactivity, and clinical outcomes

**Figure 3.**
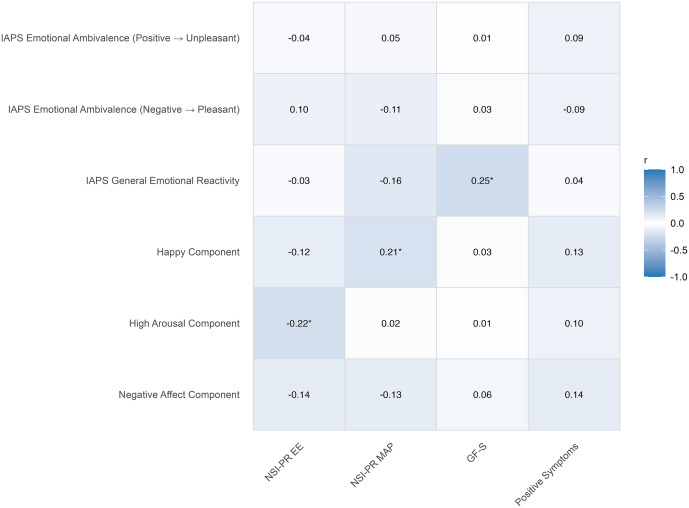
Correlation matrix between FaceReader components, IAPS emotional reactivity components, and clinical outcomes (uncorrected). Facial emotion predictors include three FaceReader principal components reflecting negative affect, high arousal, and happy facial expressions. IAPS predictors include three components reflecting general emotional reactivity and emotional ambivalence, distinguished by incongruent negative emotion to pleasant stimuli and incongruent positive emotion to unpleasant stimuli. Outcome measures include current social functioning (GFS-S), emotional expressivity (NSI-PR EE), motivation and pleasure (NSI-PR MAP), and total positive symptom severity (SIPS). Cell values indicate Pearson’s *r.* An asterisk indicates *p* < .05. Figure 3. long description.

Figure 3 presents correlations among facial expression components, emotional reactivity components, clinical symptoms, and social functioning within the CHR sample. Facial expression components showed minimal associations with emotional reactivity components and positive symptom severity, but stronger relationships with negative symptoms and social functioning.

Lower scores on the high-arousal facial expression component were associated with greater NSI-PR EE (*r* = −.22, *p* = .027), such that reduced arousal was related to more severe expressive impairment (see Supplementary Figure S5A). In contrast, higher scores on the happy facial emotion component were associated with greater NSI-PR MAP (*r* = .21, *p* = .044), such that greater happy facial expression was associated with more severe motivation and pleasure impairment (see Supplementary Figure S5B). These effects were modest and did not survive FDR correction.

Among emotional reactivity components, greater general emotional reactivity was associated with better current social functioning (*r* = .25, *p* = .028), such that stronger positive and negative emotional responses to pleasant and unpleasant stimuli were associated with better social functioning (see Supplementary Figure S6). However, this effect did not survive FDR correction. No other emotional reactivity components were significantly associated with negative symptom domains or positive symptom severity.

### Incremental contribution of facial expression components

Results of the two-step sequential regression analyses examining whether facial emotion components explained variance in clinical and functional outcomes beyond emotional reactivity are presented in Table 2.

**Table 2.** Two-step regression analyses examining whether FaceReader components explain variance in clinical outcomes over and above emotional reactivity Table 2. long description.

Model fit measures for NSI-PR emotional expressivity							
Model	Predictors	*R* ^2^	Adj *R* ^2^	*F*	df1	df2	*p*
1	Sex, Age, IAPS Emotional Ambivalence	0.033	−0.007	0.96	3	72	.49
2	Sex, Age, IAPS, High Arousal Component	0.050	0.003	0.94	4	71	.45

Model comparison					Δ *R* ^2^	ΔF	df1	df2	*p*
Model 1	Model 2				0.017	1.28	1	71	.26

Model fit measures for NSI-PR motivation and pleasure							
Model	Predictors	*R* ^2^	Adj *R* ^2^	*F*	df1	df2	*p*
1	Sex, Age, IAPS General Emotional Reactivity	0.063	0.023	1.59	3	71	.20
2	Sex, Age, IAPS, Happy Component	0.152	0.104	3.14	4	70	.02

Model Comparison					Δ *R* ^2^	ΔF	df1	df2	*p*
Model 1	Model 2				0.089	7.38	1	70	.008*

Model fit measures for GFS-S current social functioning							
Model	Predictors	*R* ^2^	Adj *R^2^*	*F*	df1	df2	*p*
1	Sex, Age, IAPS General Emotional Reactivity	0.097	0.059	2.58	3	72	.060
2	Sex, Age, IAPS, Negative Valence Component	0.104	0.053	2.06	4	71	.096

Model comparison					Δ *R* ^2^	ΔF	df1	df2	*p*
Model 1	Model 2				0.007	0.53	1	71	.470

*Note:* Models were adjusted for participant sex and age and conducted separately for each outcome. In Step 1, participant sex, age, and a single IAPS emotional reactivity principal component were entered as predictors. Specifically, NSI-PR EE models included the IAPS emotional ambivalence component reflecting negative emotions to pleasant stimuli, whereas NSI-PR MAP and GFS-S models included the IAPS general emotional reactivity component. In Step 2, one FaceReader component was added to each model based on the correlation matrix (for NSI-PR EE, the high arousal component was added; for NSI-PR MAP, the happy expression component was added; and for GFS-S, the negative valence component was added). Δ*R* ^2^ and Δ*F* indicate the added contribution of the FaceReader component beyond IAPS emotional reactivity, adjusted for sex and age; *p* values correspond to the Δ*F* test.

For NSI-PR EE, the Step 1 model including sex, age, and the IAPS emotional ambivalence component reflecting negative emotions to pleasant stimuli was non-significant (*R* ^2^ = 0.033, *p* = .49). Adding the high-arousal facial emotion component in Step 2 did not significantly improve model fit (ΔR^2^ = 0.017, ΔF = 1.28, *p* = .26), indicating that facial expressions did not explain additional variance in emotional expressivity beyond emotional reactivity.

For NSI-PR MAP, the Step 1 model including sex, age, and the IAPS general emotional reactivity component accounted for a small proportion of variance but was not statistically significant (*R* ^2^ = 0.063, *p* = .20). Adding the happy facial emotion component in Step 2 significantly improved model fit, accounting for an additional 8.9% of the variance (ΔR^2^ = 0.089, ΔF = 7.38, *p* = .008; FDR-corrected *p* = .024). This effect remained significant in sensitivity analyses with assessor included as a random intercept (χ^2^ = 6.33, *p* = .012; FDR-corrected *p* = .036; see Supplementary Table S6).

For current social functioning, the Step 1 model that included sex, age, and the IAPS general emotional reactivity component accounted for nearly 10% of the variance, although the model did not reach significance (*R* ^2^ = 0.097, *p* = .06). Adding the negative-valence facial emotion component in Step 2 did not significantly improve model fit (ΔR^2^ = 0.007, ΔF = 0.53, *p* = .47).

## Discussion

This study examined whether objective facial expression and subjective emotional reactivity capture overlapping or distinct aspects of negative symptoms in CHR individuals. Facial expression and emotional reactivity showed largely non-overlapping associations, suggesting that they reflect partially distinct processes in CHR. These findings extend prior work in schizophrenia and indicate that dissociations between emotional expression and experience may already be present in psychosis-risk populations.

CHR individuals differed from HCs in facial expression only for disgust, with no significant group differences observed for happy, sad, neutral, or angry expressions. Although this pattern was unexpected given our prediction of reduced positive facial expression in CHR, it aligns with prior evidence suggesting that expressive alterations in CHR may be emotion specific rather than globally reduced, with some negative facial expressions, such as anger, sadness, and fear, preserved or heightened (Cowan et al., 2022; Gupta et al., 2019). Given that facial expressions were examined during a relatively neutral interview context, elevated disgust may reflect broader changes in how emotion is expressed in CHR individuals rather than genuine disgust. We did not observe meaningful associations between facial expression measures and disorganization symptoms, suggesting that these effects are unlikely to be driven by disorganization. The direction of this effect remained stable in sensitivity analyses, although it did not remain significant after correction.

Consistent with prior work, CHR participants also showed altered emotional reactivity during the IAPS task. CHR participants reported greater positive emotion ratings to unpleasant stimuli, which was the only effect that survived FDR correction. Nominal group differences were also observed for reduced positive emotion in response to pleasant stimuli, lower negative emotion ratings to unpleasant stimuli, and lower arousal ratings to pleasant stimuli, although these effects did not survive correction. Together, these patterns are consistent with prior evidence of altered emotional responding across both pleasant and unpleasant stimuli in psychosis-risk populations (Riehle et al., 2024).

To characterize the dimensional structure of facial expression and emotional reactivity in CHR participants, we examined PCA-derived components from FaceReader and IAPS. Facial expression variables yielded a three-component structure, reflecting negative-valence expression, high-arousal expression, and happy expression. Rather than supporting a positive-versus-negative emotion distinction, this pattern suggests that facial behavior in CHR may be organized around broader dimensions, particularly valence and arousal (Cohen et al., 2013; Cowan et al., 2022). This dimensional structure may be especially relevant for understanding negative symptoms, as prior work in CHR has shown that clinician-rated expressive deficits are more strongly associated with reductions in high-arousal expressions such as fear and surprise (Cowan et al., 2022). These findings suggest that arousal-based dimensions of facial behavior may be particularly informative for characterizing expressive impairment in psychosis-risk populations.

PCA of IAPS emotional reactivity also yielded a three-component structure, including a general emotional reactivity component and two components reflecting emotional ambivalence. The general reactivity component captured congruent emotional responding (i.e. positive emotion to pleasant stimuli and negative emotion to unpleasant stimuli), whereas the emotional ambivalence components reflected incongruent responses, characterized by negative emotion to pleasant stimuli and positive emotion to unpleasant stimuli. This suggests that emotional experience in CHR may reflect reduced specificity or differentiation, rather than simple attenuation. Altered congruent reactivity is consistent with prior findings in psychosis-risk populations (Gruber et al., 2018; Strauss et al., 2018), whereas the emotional ambivalence components align with schizophrenia research demonstrating mixed or overlapping emotional responses to evocative stimuli and simultaneous activation of positive and negative emotional systems (Strauss et al., 2017; Trémeau et al., 2009).

Notably, emotional reactivity components showed weak associations with facial expression components, indicating that subjective emotional experience and outward facial expression are partially independent, consistent with prior work in schizophrenia (Berenbaum & Oltmanns, 1992; Kring et al., 1993; Kring & Moran, 2008). Emotional reactivity was also unrelated to motivational or expressive negative symptoms, although it showed a modest association with social functioning. Together, these findings suggest that motivational and expressive impairments are not fully explained by reduced in-the-moment pleasure or emotional intensity, consistent with models distinguishing consummatory pleasure from motivational processes (Cohen et al., 2010; Gard et al., 2007).

Facial expression components showed selective, rather than global, associations with negative symptom domains. Associations with positive symptom severity were minimal, underscoring the specificity of facial expression to negative symptoms specifically rather than global illness severity (Fulford et al., 2013). Lower high-arousal facial expression was associated with greater clinician-rated emotional expressivity deficits, consistent with prior evidence that expressive impairment may be especially related to reductions in high-arousal facial behaviors, such as fear and surprise, rather than broad suppression of all facial affect (Cowan et al., 2022). In contrast, greater happy facial expression was associated with more severe motivation and pleasure impairment. Although modest and not significant after correction, this pattern challenges the assumption that outwardly positive facial behavior reflects intact emotional experience. One possible interpretation is that positive facial behavior may not index genuine positive emotional experience, rather socially learned, context-dependent, or non-genuine affiliative behavior (Ricard et al., 2022). This highlights the importance of assessing expressive and experiential domains independently.

The regression analyses further suggest that facial expression may provide incremental information beyond emotional reactivity in relation to some negative symptom domains. Specifically, the happy facial expression component explained an additional 8.9% of variance in motivation and pleasure, with the full model explaining 15.2% of variance overall. Although this effect remained significant in sensitivity analyses, the magnitude of explained variance was modest and the direction of association was unexpected, such that greater happy facial expression predicted more severe motivation and pleasure impairment. These findings should therefore be interpreted cautiously. Facial expression did not explain additional variance in emotional expressivity or social functioning, suggesting that its incremental utility may be selective rather than general. Automated facial expression analysis may offer a complementary, rather than redundant, approach to assessing negative symptom domains.

Several limitations should be considered. First, facial expressions were derived from brief, naturalistic segments of a clinical interview rather than from emotionally evocative tasks. Although this approach increased ecological validity, it may also have reduced sensitivity to group differences. Prior work has demonstrated that even brief interview segments contain sufficient information to detect meaningful differences in facial behavior (Cohen et al., 2013; Gupta et al., 2019, 2022). Second, emotional reactivity and facial expression were not assessed in response to the same stimuli, unlike emotion-induction paradigms commonly used in schizophrenia research (Kring et al., 1993; Kring & Neale, 1996). Future studies would benefit from directly comparing these domains within the same context. Third, the cross-sectional design limits conclusions about temporal relationships among emotional expressivity, emotional experience, and negative symptoms. Fourth, medication use was more common in the CHR group and included a range of psychiatric medications that may influence facial behavior, although the overall pattern of findings remained robust after controlling for antipsychotic use. Automated facial analysis tools may be subject to demographic bias, highlighting the need for continued validation across diverse populations (Rhue, 2018). Finally, because there is no single gold-standard criterion for emotional expression or emotional reactivity in psychosis-risk research, associations among automated facial expressions, emotional reactivity measures, and clinician-rated symptoms should be interpreted as construct-level convergence or divergence rather than definitive validation of any single measure.

Overall, this study shows that automated facial expression analysis may offer a scalable and objective complement to existing approaches for assessing negative symptoms in CHR individuals. These findings do not indicate that facial expression alone is sufficient to characterize negative symptoms in psychosis risk, but rather that automated facial behavior may provide modest but unique information that is not fully captured by self-reported emotional reactivity. When integrated with existing approaches and combined with linguistic and acoustic features, automated facial analysis may help refine the measurement of negative symptom domains and improve the precision of multimodal assessment in psychosis-risk populations (Bilgrami et al., 2025; Gupta et al., 2022; Lozano-Goupil et al., 2025).

## Supporting information

10.1017/S0033291726104826.sm001 Bertrand et al. supplementary material Bertrand et al. supplementary material

## Supplementary material

The supplementary material for this article can be found at http://doi.org/10.1017/S0033291726104826.

## Table 1. Long description

Beginning at the top, the table lists group columns: HC with N equals 41, CHR with N equals 101, and a statistics column. The first section covers age, showing mean age for HC as 22.56 years with standard deviation 4.02, and CHR as 23.35 years with standard deviation 3.79. Welch’s t-test for age is t open parenthesis 71.65 close parenthesis equals minus 1.08, p equals point 29. Sex is next, with male HC 14 (34.1 percent), female HC 27 (65.9 percent), male CHR 42 (41.6 percent), female CHR 59 (58.4 percent), chi-squared open parenthesis 1 close parenthesis equals 0.68, p equals point 41. Education follows, mean years for HC 14.59 (2.30), CHR 14.22 (1.84), t open parenthesis 62.90 close parenthesis equals 0.89, p equals point 38, missing values: HC 0, CHR 5 (4.9 percent). Ethnicity is listed: Hispanic HC 4 (9.8 percent), CHR 10 (9.9 percent), Not Hispanic HC 37 (90.0 percent), CHR 91 (90.1 percent), chi-squared open parenthesis 1 close parenthesis equals 0.00, p equals point 98. Race categories: Asian HC 16 (39.0 percent), CHR 15 (14.9 percent); American Indian HC 0, CHR 1 (1.0 percent); Black HC 2 (4.9 percent), CHR 14 (13.9 percent); White HC 22 (53.7 percent), CHR 52 (51.5 percent); More than one race HC 1 (2.4 percent), CHR 13 (12.9 percent); missing HC 0, CHR 6 (5.9 percent); chi-squared open parenthesis 1 close parenthesis equals 0.002, p equals point 96. Medication section: psychiatric medication HC 0, CHR 34 (33.7 percent); antidepressant HC 0, CHR 21 (20.8 percent); antianxiety HC 0, CHR 6 (5.9 percent); antipsychotic HC 0, CHR 8 (7.9 percent); stimulant HC 0, CHR 15 (14.9 percent); mood stabilizer HC 0, CHR 6 (5.9 percent); missing HC 4 (9.8 percent), CHR 9 (8.9 percent); Fisher’s p less than point 001. Current diagnoses: major depressive disorder HC 0, CHR 16 (15.8 percent); persistent depressive disorder HC 0, CHR 20 (19.8 percent); generalized anxiety disorder HC 0, CHR 46 (45.5 percent); social anxiety disorder HC 0, CHR 49 (48.5 percent); bipolar disorder HC 0, CHR 15 (14.8 percent); post-traumatic stress disorder HC 0, CHR 21 (20.8 percent); attention-deficit/hyperactivity disorder HC 0, CHR 34 (33.7 percent); obsessive compulsive disorder HC 0, CHR 23 (22.8 percent); substance use disorder HC 0, CHR 26 (25.7 percent); Fisher’s p less than point 001. Clinical measures: S I P S total positive HC 1.45 (1.45), CHR 10.40 (3.47), t open parenthesis 139.72 close parenthesis equals minus 21.62, p less than point 001; G F S dash S current social HC 8.59 (0.84), CHR 7.29 (1.31), t open parenthesis 110.73 close parenthesis equals 6.95, p less than point 001; N S I dash P R E E HC 2 (1.41), CHR 3.05 (3.82), t open parenthesis 5.06 close parenthesis equals minus 1.31, p equals point 25; N S I dash P R M A P HC 7 (2.83), CHR 11.30 (7.75), t open parenthesis 4.09 close parenthesis equals minus 2.83, p equals point 046.

Navigate back to Table 1..

## Figure 1. Long description

Starting from the top-left and moving right, the grid contains panels for Happy, Neutral, Sad (top row), Angry, Disgusted, Scared (middle row), Surprised, Valence, and Arousal (bottom row). Each panel plots mean emotion scores on the y-axis and group (chr in blue, hc in gray) on the x-axis. In all panels, individual data points are shown with group means and 95 percent confidence intervals as black error bars. For Happy, Neutral, Sad, Angry, Scared, Surprised, Valence, and Arousal, mean scores are similar between chr and hc groups. In the Disgusted panel (middle row, center), the chr group shows a visibly lower mean score than the hc group, marked with an asterisk indicating statistical significance. The legend at the right identifies chr as blue and hc as gray.

Navigate back to Figure 1..

## Figure 2. Long description

From top left to bottom right, the chart is arranged in a three-by-three grid. Columns represent stimulus categories: pleasant, neutral, and unpleasant. Rows represent rating types: positive, negative, and arousal. Each panel displays two groups on the x-axis: C H R (left, blue) and H C (right, gray). The y-axis in all panels is labeled self-reported rating, ranging from 1 to 5. Each participant is shown as a dot, with group means marked by a larger circle and horizontal error bars indicating 95 percent confidence intervals. In the top row (positive ratings), C H R and H C groups both rate pleasant stimuli highly, with means near 4, but H C is slightly higher. For neutral stimuli, both groups rate around 3. For unpleasant stimuli, C H R group rates higher than H C, with a significant difference marked by three asterisks next to the C H R mean. In the middle row (negative ratings), both groups rate pleasant and neutral stimuli low, around 1 to 2, with no clear group difference. For unpleasant stimuli, both groups rate higher, but H C is slightly higher than C H R. In the bottom row (arousal ratings), both groups rate pleasant and unpleasant stimuli around 3 to 4, with H C slightly higher for pleasant stimuli. For neutral stimuli, both groups rate lower, around 2. No other significant group differences are visually indicated.

Navigate back to Figure 2..

## Figure 3. Long description

From left to right, columns are labeled N S I dash P R E E, N S I dash P R M A P, G F dash S, and Positive Symptoms. From top to bottom, rows are labeled I A P S Emotional Ambivalence open parenthesis Positive approaches Unpleasant close parenthesis, I A P S Emotional Ambivalence open parenthesis Negative approaches Pleasant close parenthesis, I A P S General Emotional Reactivity, Happy Component, High Arousal Component, and Negative Affect Component. Each cell contains a Pearson r value. Significant correlations are indicated by an asterisk. Notable values include a negative correlation of minus zero point two two asterisk between High Arousal Component and N S I dash P R E E, a positive correlation of zero point two one asterisk between Happy Component and N S I dash P R M A P, and a positive correlation of zero point two five asterisk between I A P S General Emotional Reactivity and G F dash S. The color bar on the right maps r values from negative one to one, with darker blue for negative and lighter blue for positive correlations.

Navigate back to Figure 3..

## Table 2. Long description

Starting from the top, the first table presents model fit measures for N S I dash P R emotional expressivity. Step 1 predictors are sex, age, and I A P S emotional ambivalence, yielding R squared 0.033, adjusted R squared minus 0.007, F 0.96, degrees of freedom 3 and 72, p 0.49. Step 2 adds the high arousal component, with R squared 0.050, adjusted R squared 0.003, F 0.94, degrees of freedom 4 and 71, p 0.45. Model comparison shows delta R squared 0.017, delta F 1.28, degrees of freedom 1 and 71, p 0.26. The second table details N S I dash P R motivation and pleasure. Step 1 predictors are sex, age, and I A P S general emotional reactivity, R squared 0.063, adjusted R squared 0.023, F 1.59, degrees of freedom 3 and 71, p 0.20. Step 2 adds the happy component, R squared 0.152, adjusted R squared 0.104, F 3.14, degrees of freedom 4 and 70, p 0.02. Model comparison yields delta R squared 0.089, delta F 7.38, degrees of freedom 1 and 70, p 0.008. The third table shows G F S dash S current social functioning. Step 1 predictors are sex, age, and I A P S general emotional reactivity, R squared 0.097, adjusted R squared 0.059, F 2.58, degrees of freedom 3 and 72, p 0.060. Step 2 adds the negative valence component, R squared 0.104, adjusted R squared 0.053, F 2.06, degrees of freedom 4 and 71, p 0.096. Model comparison gives delta R squared 0.007, delta F 0.53, degrees of freedom 1 and 71, p 0.470. All models are adjusted for sex and age, and delta R squared and delta F indicate the incremental contribution of FaceReader components beyond I A P S emotional reactivity.

Navigate back to Table 2..
