# Supplementary material for: Computerized assessments of emotional expression and emotional reactivity predict negative symptoms in individuals at clinical high-risk for psychosis
Source: Psychol Med. 2026 Jun 10;56:e184. doi: 10.1017/S0033291726104826 (PMC13280694; doi:10.1017/S0033291726104826)
Supplement: Bertrand et al. supplementary material [file S0033291726104826sup001.zip › Supplementary Materials_CLEAN.docx]

**Supplementary Materials**

Figure S1: Analytic pipeline

Table S1: Descriptive statistics and effect sizes for emotional expression by group

Figure S2: FaceReader facial emotion differences across groups, adjusted for sex, age, antipsychotic use, with assessor as a random intercept

Table S2: Assessor-related variance in facial emotion expressions

Table S3: Group differences in raw ratings from the International Affective Picture System (IAPS)

Figure S3: Scree plots for principal component analysis of FaceReader variables in the CHR sample

Table S4: Pattern matrix from principal component analysis for FaceReader

Figure S4: Scree plots for principal component analysis of IAPS variables in the CHR sample

Table S5: Pattern matrix from principal component analysis for IAPS

Figure S5: Correlations between facial expression components and negative symptoms in the CHR sample (uncorrected).

Figure S6. Association between IAPS general emotional reactivity and social functioning

Table S6: Two-step sequential mixed-effects regression of outcome measures, adjusted for sex and age, with assessor included as a random intercept

Figure S1: Analytic pipeline

Fig S1. *Analytic pipeline*. Aim 1: Group differences in facial expression and emotional reactivity across the full sample; Aim 2: Principal components analysis (PCA) was conducted separately on FaceReader and IAPS variables within the CHR group; Aim 3: associations between PCA-derived components and clinical outcomes were examined using Pearson correlations; and Aim 4: two-step sequential regressions tested whether facial expression components explained variance in negative symptoms beyond emotional reactivity. Sample sizes vary across analyses due to missing data. CHR = clinical high risk; HC = healthy control; NSI-PR EE = Negative Symptom Inventory–Psychosis Risk emotional expressivity; NSI-PR MAP = motivation and pleasure; GFS-S = Global Functioning Scale–Social; SIPS = Structured Interview for Psychosis-Risk Syndromes; IAPS = International Affective Picture System; PCA = principal components analysis.

Table S1: Descriptive statistics and effect sizes for emotional expression by group

| **Emotion** | **CHR Mean (SD)** | **HC Mean (SD)** | **Hedges’ g** | **95% CI** |
| --- | --- | --- | --- | --- |
| Angry | 0.038 (0.061) | 0.032 (0.040) | 0.12 | [−0.24, 0.48] |
| Arousal | 0.359 (0.063) | 0.347 (0.062) | 0.18 | [−0.18, 0.54] |
| **Disgusted** | 0.037 (0.053) | 0.019 (0.017) | **0.40** | **[0.03, 0.76]** |
| Happy | 0.078 (0.081) | 0.066 (0.108) | 0.14 | [−0.22, 0.50] |
| Neutral | 0.676 (0.166) | 0.710 (0.160) | −0.21 | [−0.57, 0.15] |
| Sad | 0.078 (0.084) | 0.074 (0.069) | 0.06 | [−0.30, 0.42] |
| Scared | 0.027 (0.040) | 0.026 (0.041) | 0.02 | [−0.34, 0.38] |
| Surprised | 0.061 (0.079) | 0.054 (0.053) | 0.09 | [−0.28, 0.45] |
| Valence | −0.055 (0.129) | −0.042 (0.144) | −0.09 | [−0.46, 0.27] |

Table S1. *Descriptive statistics and effect sizes for emotional expression by group.* Values represent group means and standard deviations across each emotion and group. Hedges’ g reflects standardized mean differences between groups (CHR - HC), with positive values indicating higher scores in the CHR group and negative values indicating higher scores in the HC group. Confidence intervals represent 95% intervals around Hedges’ g.

Figure S2: FaceReader facial emotion differences across groups, adjusted for sex, age, antipsychotic use, with assessor as a random intercept


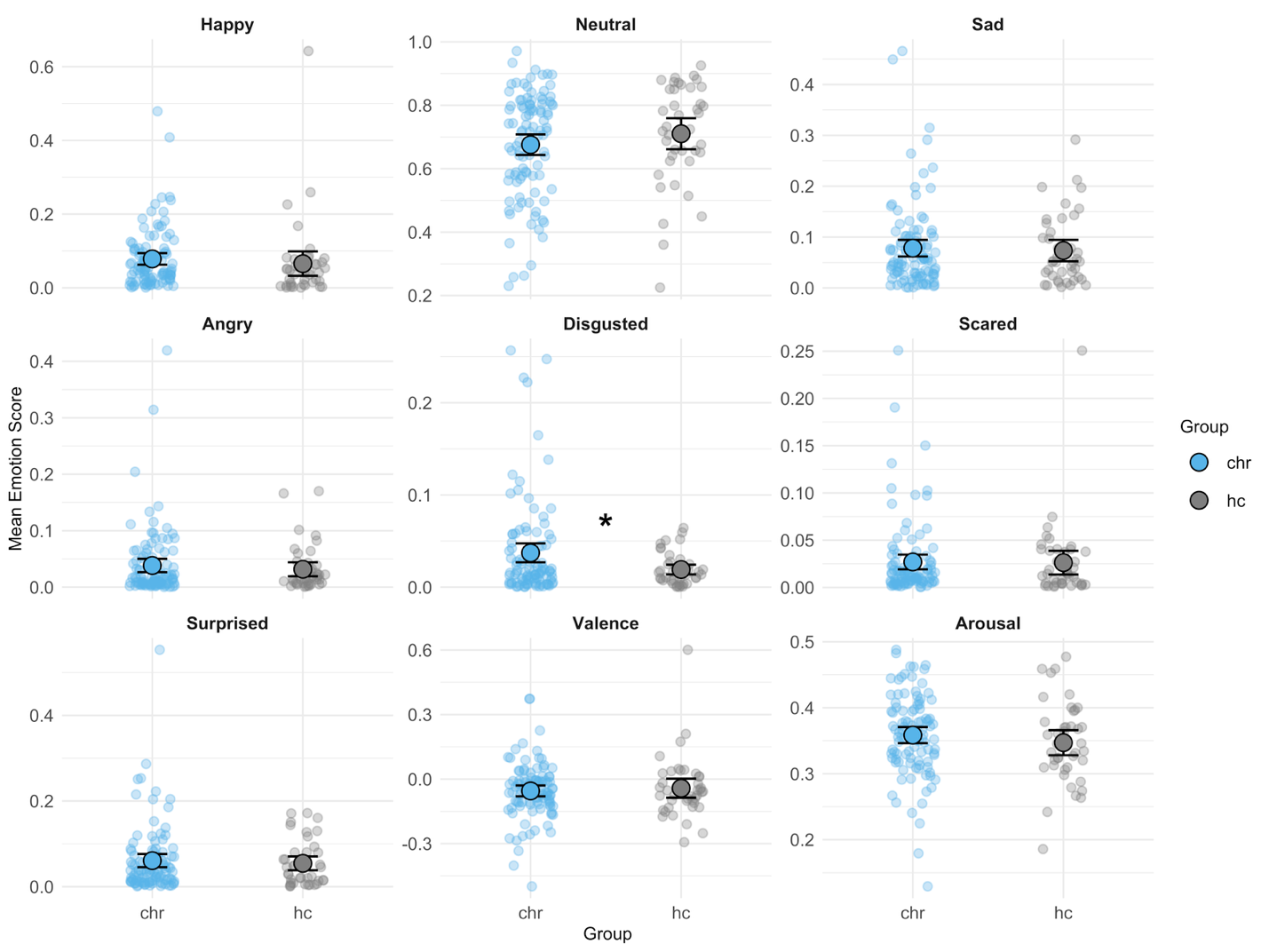


Fig S2. *FaceReader-derived facial emotion differences between groups, adjusted for sex, age, and antipsychotic medication use, with assessor included as a random intercept.* A nominal group difference was observed for Disgusted (p = .026), whereas no other emotions showed significant group differences (p > .07). Significant main effects of sex were observed for Happy (p = .001), Arousal (p = .006), and Neutral (p = .014). No main effects of age were significant for any emotion (p > .25). No group effects survived false discovery rate (FDR) correction. Error bars represent 95% confidence intervals. Asterisks denote nominal group effects (p < .05).

Table S2: Assessor-related variance in facial emotion expressions

| **Emotion** | **ICC (Assessor)** | **Assessor Variance** | **Residual Variance** |
| --- | --- | --- | --- |
| Neutral | 0.13 | 0.0036 | 0.0230 |
| Happy | 0.21 | 0.0017 | 0.0062 |
| Sad | 0.08 | 0.0005 | 0.0059 |
| Angry | 0.02 | 0.0001 | 0.0030 |
| Surprised | 0.17 | 0.0010 | 0.0045 |
| Scared | 0.04 | 0.0001 | 0.0016 |
| Disgusted | 0.00 | <0.00001 | 0.0021 |
| Valence | 0.11 | 0.0019 | 0.0160 |
| Arousal | 0.00 | 0.0000 | 0.0037 |

Table S2. *Assessor-related variance components for facial emotion expressions. Values* are derived from mixed-effects models including group, sex, and age as fixed effects and assessor as a random intercept. The intraclass correlation coefficient (ICC) reflects the proportion of total variance attributable to assessor. Assessor variance represents between-assessor variability, whereas residual variance reflects within-assessor and participant-level variability not explained by the model.

Table S3: Group differences in raw ratings from the International Affective Picture System (IAPS)

| **IAPS Variables** | **CHR Mean (SD)** | **HC Mean (SD)** | **Hedges’ g** | **95% CI** |
| --- | --- | --- | --- | --- |
| Negative Pleasantness | 1.25 (0.33) | 1.07 (0.12) | 0.60 | [0.21, 0.99] |
| Negative Unpleasantness | 3.78 (0.78) | 4.06 (0.51) | -0.39 | [-0.77, 0.00] |
| Positive Arousal | 3.31 (0.73) | 3.57 (0.53) | -0.38 | [-0.77, 0.00] |
| Positive Pleasantness | 3.75 (0.77) | 4.00 (0.57) | -0.35 | [-0.69, -0.01] |
| Positive Unpleasantness | 1.44 (0.35) | 1.31 (0.37) | 0.35 | [-0.04, 0.73] |
| Neutral Pleasantness | 2.00 (0.73) | 1.80 (0.57) | 0.29 | [-0.09, 0.68] |
| Neutral Arousal | 2.28 (0.66) | 2.44 (0.47) | -0.25 | [-0.63, 0.14] |
| Negative Arousal | 3.19 (0.69) | 3.35 (0.66) | -0.23 | [-0.62, 0.15] |

Table S3. *Group differences in raw ratings from the International Affective Picture System (IAPS).* Values represent group means and standard deviations across each emotion and group. Hedges’ g reflects standardized mean differences between groups (CHR - HC), with positive values indicating higher scores in the CHR group and negative values indicating higher scores in the HC group. Confidence intervals represent 95% intervals around Hedges’ g.

Figure S3: Scree plots for principal component analysis of FaceReader variables in the CHR sample


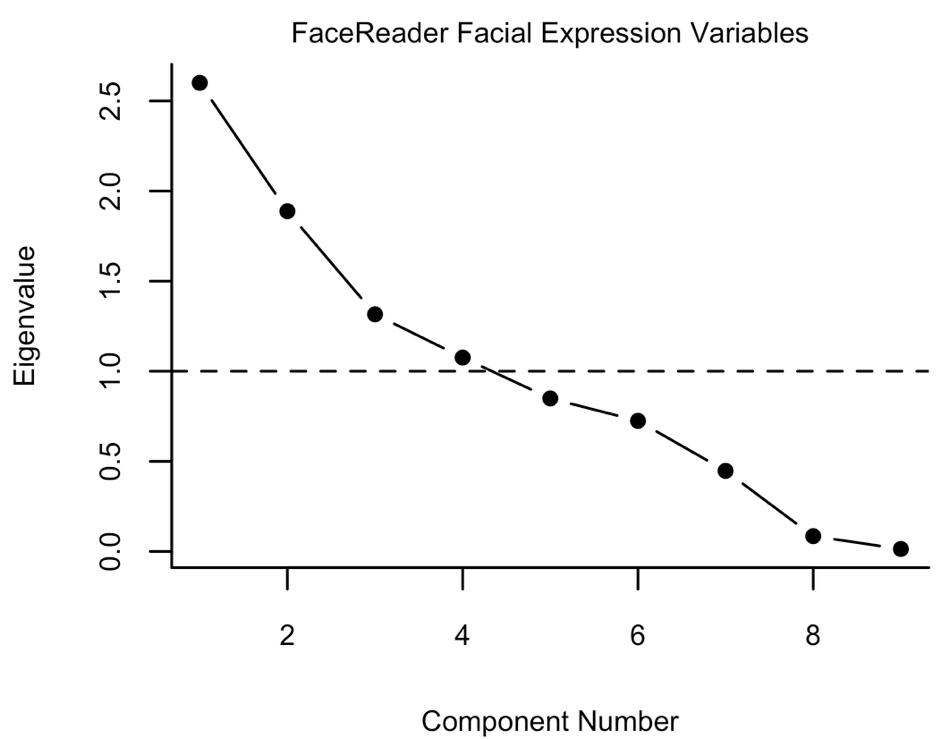


Figure S3: *Scree plots for principal component analysis of FaceReader variables in the CHR sample.* Although four components had eigenvalues greater than 1.0, a clear inflection in after the third component, supporting the retention of three components. The dashed horizontal line indicates the Kaiser criterion.

Table S4: Pattern matrix from principal component analysis for FaceReader

| **FaceReader** | **Negative Affect (PC1)** | **High Arousal (PC2)** | **Happy (PC3)** |
| --- | --- | --- | --- |
| Valence | 0.91 |  | 0.43 |
| Sad | -0.76 |  |  |
| Angry | -0.60 |  |  |
| Happy |  |  | 0.91 |
| Neutral | 0.54 |  | -0.67 |
| Disgusted |  |  |  |
| Scared | -0.44 | 0.79 |  |
| Arousal |  | 0.68 |  |
| Surprised |  | 0.55 |  |

Table S4. *Pattern matrix from principal component analysis (PCA) of FaceReader emotion variables using Promax rotation.* Values represent standardized pattern loadings, loadings < .40 are removed for clarity. Principal Component 1 was characterized by strong positive loadings for valence, and strong negative loadings for sad and angry expressions (negative affect component). Principal Component 2 showed strong positive loadings for scared and surprised expressions (high arousal component). Principal Component 3 loaded predominantly on happy expression (happy component).

Figure S4: Scree plots for principal component analysis of IAPS variables in the CHR sample


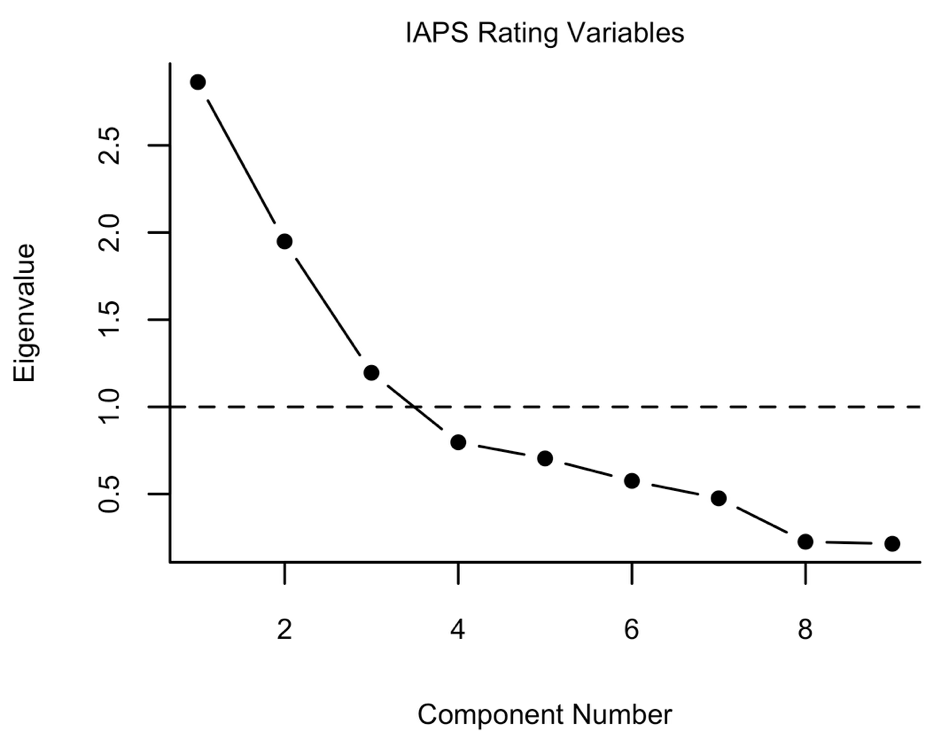


Figure S4. *Scree plots for principal component analysis of IAPS variables in the CHR sample.* Three components exceeded an eigenvalue of 1.0, which supported a three-component solution. The dashed horizontal line indicates the Kaiser criterion.

Table S5: Pattern matrix from principal component analysis for IAPS

| **IAPS Variable** | **General Emotional Reactivity (PC1)** | **Emotional Ambivalence (PC2) Negative Emotion to Pleasant Stimuli** | **Emotional Ambivalence (PC3) Positive Emotion to Unpleasant Stimuli** |
| --- | --- | --- | --- |
| Positive Pleasant | 0.879 |  |  |
| Positive Arousal | 0.698 |  |  |
| Positive Unpleasant |  | 0.847 |  |
| Neutral Pleasant |  |  | 0.768 |
| Neutral Arousal |  | 0.618 |  |
| Neutral Unpleasant |  | 0.786 |  |
| Negative Pleasant |  |  | 0.794 |
| Negative Arousal | 0.689 |  |  |
| Negative Unpleasant | 0.878 |  |  |

Table S5. *Pattern matrix from principal component analysis (PCA) of International Affective Picture System (IAPS) task variables using Promax rotation.* Values represent standardized pattern loadings; loadings < .40 are removed for clarity. Principal Component 1 reflected general emotional reactivity, characterized by congruent positive and negative emotional responses to pleasant and unpleasant stimuli, respectively. Principal Components 2 and 3 reflected emotional ambivalence, distinguished by opposite directions of the stimulus-rating mismatch (negative emotion to pleasant stimuli for PC2 and positive emotion to unpleasant stimuli for PC3).

Figure S5: Correlations between facial expression components and negative symptoms in the CHR sample (uncorrected).


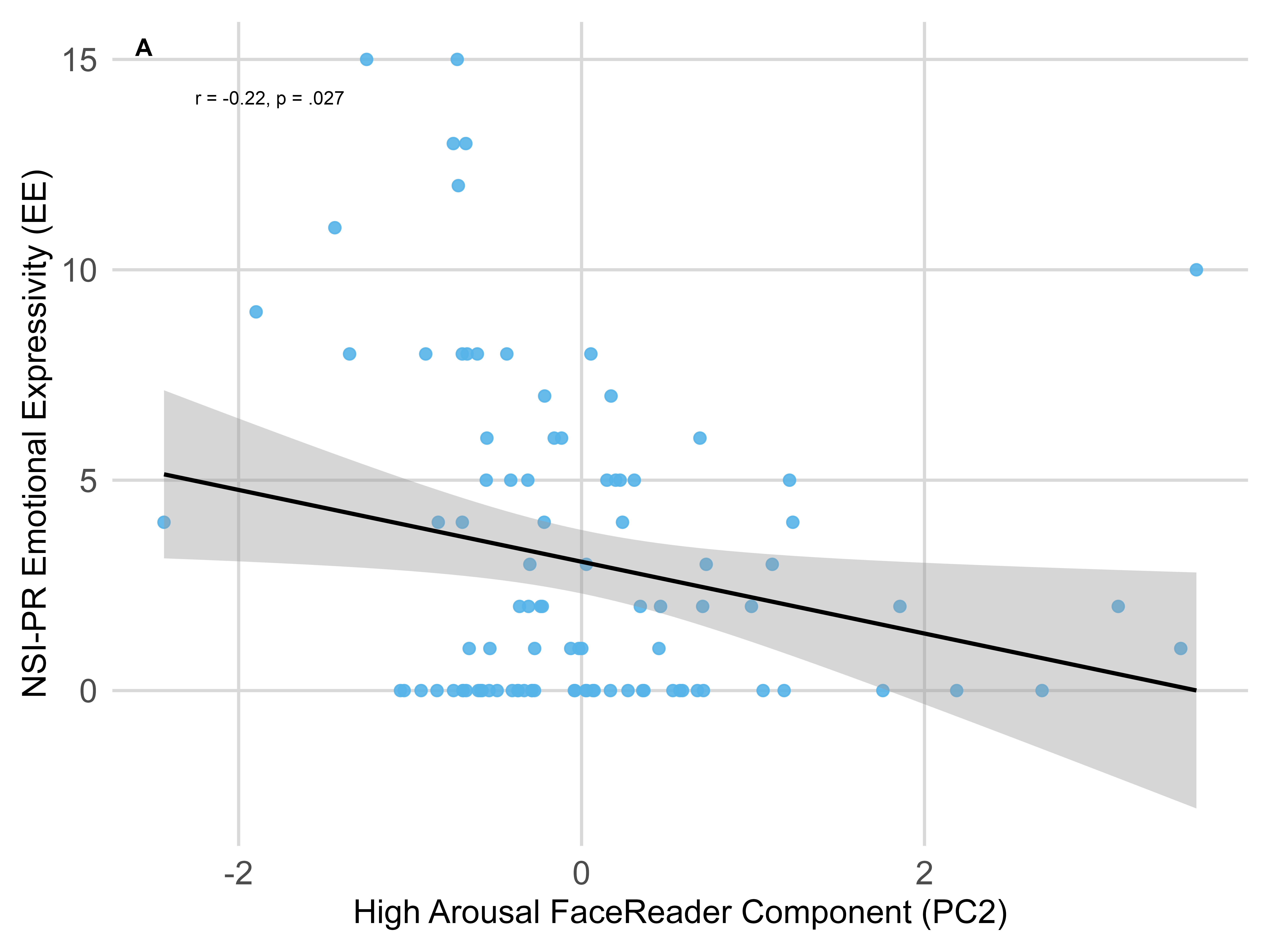


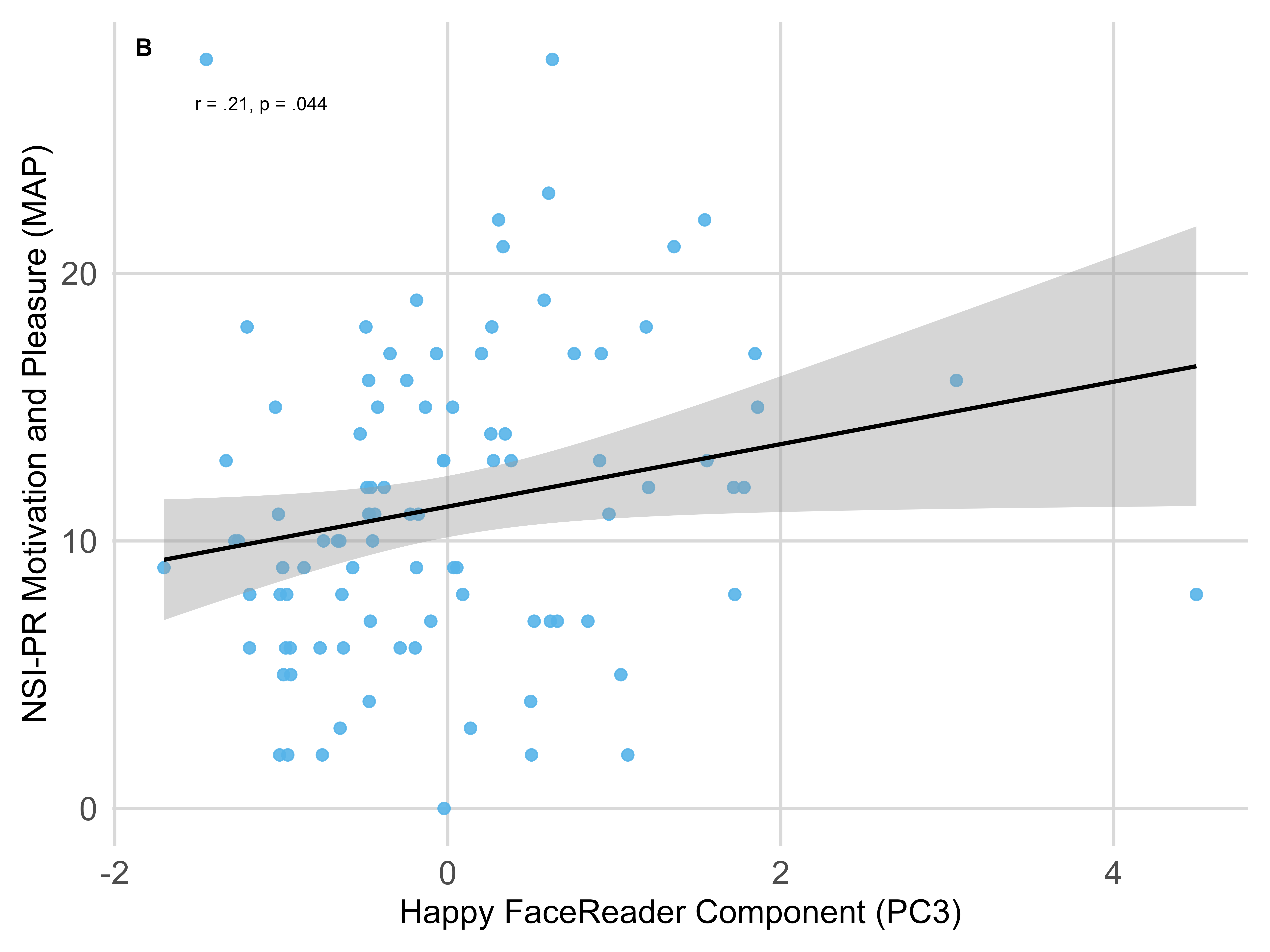


Fig S5. *Correlations between facial expression components and negative symptoms in the CHR sample (uncorrected).* (A) Lower scores on the FaceReader High Arousal component were associated with higher NSI-PR EE scores, indicating greater expressivity impairment (p = .027). (B) Higher scores on the FaceReader Happy component were associated with higher NSI-PR MAP scores, indicating greater motivation/pleasure impairment (p = .044).

B

Figure S6. Association between IAPS general emotional reactivity and social functioning


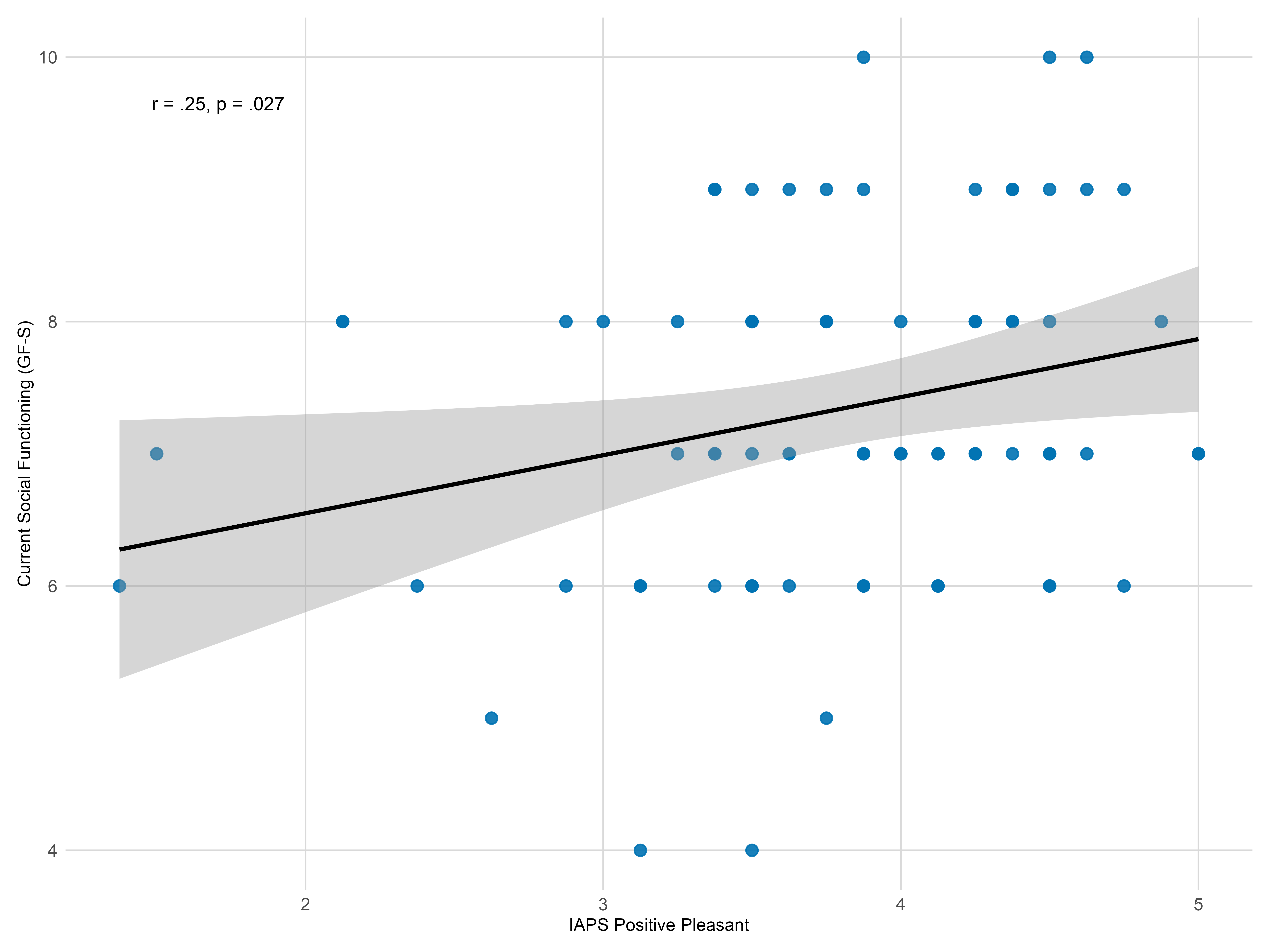


Fig S6. *Association between IAPS general emotional reactivity and social functioning.* Higher scores on the general emotional reactivity component (reflecting stronger congruent emotional responses to pleasant and unpleasant stimuli) were associated with higher current social functioning (r = .25, p = .028; uncorrected).

Table S6: Two-step sequential mixed-effects regression of outcome measures, adjusted for sex and age, with assessor included as a random intercept

| Model Fit Measures for NSI-PR Emotional Expressivity | | | | |
| --- | --- | --- | --- | --- |
| Model | Predictors | χ² | df | p |
| 1 | Sex, Age, IAPS General Emotional Reactivity | - | - | - |
| 2 | Sex, Age, IAPS, High Arousal Component | 2.10 | 1 | .15 |

| Model Fit Measures for NSI-PR Motivation and Pleasure | | | | |
| --- | --- | --- | --- | --- |
| Model | Predictors | χ² | df | p |
| 1 | Sex, Age, IAPS Emotional Ambivalence (Negative Emotion to Pleasant Stimuli) | - | - | - |
| 2 | Sex, Age, IAPS, Happy Component | 6.33 | 1 | **.010*** |

| Model Fit Measures for GFS-S Current Social Functioning | | | | |
| --- | --- | --- | --- | --- |
| Model | Predictors | χ² | df | p |
| 1 | Sex, Age, IAPS Emotional Ambivalence (Positive Emotion to Unpleasant Stimuli) | - | - | - |
| 2 | Sex, Age, IAPS, Negative Affect Component | 0.22 | 1 | .64 |

Table S6: *Two-step sequential mixed-effects models examining whether FaceReader components explain variance in clinical outcomes over and above emotional reactivity, adjusted for sex and age, with assessor included as a random intercept.* In Step 1, participant sex and age, and a single IAPS emotional reactivity principal component were entered as fixed-effect predictors. In Step 2, one FaceReader-derived component, selected based on the correlation matrix, was added to each model. All models included assessor as a random intercept to account for rater-related variance. Model comparisons reflect the improvement in model fit associated with adding the FaceReader component over and above IAPS emotional reactivity. P values correspond to the χ² test; an asterisk denotes p < .05. After FDR correction, the association between the happy facial expression component and NSI-PR Motivation and Pleasure remained significant (p = .036).
