# Supplementary material for: Computerized assessments of emotional expression and emotional reactivity predict negative symptoms in individuals at clinical high-risk for psychosis
Source: Psychol Med. 2026 Jun 10;56:e184. doi: 10.1017/S0033291726104826 (PMC13280694; doi:10.1017/S0033291726104826)
Supplement: Bertrand et al. supplementary material [file S0033291726104826sup001.zip › Supplementary Table and Figure Captions.docx]

Supplementary Table and Figure Captions

Fig S1. *Analytic pipeline*. Aim 1: Group differences in facial expression and emotional reactivity across the full sample; Aim 2: Principal components analysis (PCA) was conducted separately on FaceReader and IAPS variables within the CHR group; Aim 3: associations between PCA-derived components and clinical outcomes were examined using Pearson correlations; and Aim 4: two-step sequential regressions tested whether facial expression components explained variance in negative symptoms beyond emotional reactivity. Sample sizes vary across analyses due to missing data. CHR = clinical high risk; HC = healthy control; NSI-PR EE = Negative Symptom Inventory–Psychosis Risk emotional expressivity; NSI-PR MAP = motivation and pleasure; GFS-S = Global Functioning Scale–Social; SIPS = Structured Interview for Psychosis-Risk Syndromes; IAPS = International Affective Picture System; PCA = principal components analysis.

Table S1. *Descriptive statistics and effect sizes for emotional expression by group. Values represent group means and standard deviations across each emotion and group.* Hedges’ g reflects standardized mean differences between groups (CHR - HC), with positive values indicating higher scores in the CHR group and negative values indicating higher scores in the HC group. Confidence intervals represent 95% intervals around Hedges’ g.

Fig S2. *FaceReader-derived facial emotion differences between groups, adjusted for sex, age, site, and antipsychotic medication use, with assessor included as a random intercept.* A nominal group difference was observed for Disgusted (p = .027), whereas no other emotions showed significant group differences (p > .07). Significant main effects of sex were observed for Happy (p = .001), Arousal (p = .006), and Neutral (p = .014). No main effects of age were significant for any emotion (p > .25). No group effects survived false discovery rate (FDR) correction. Error bars represent 95% confidence intervals. Asterisks denote nominal group effects (p < .05).

Table S2: *Assessor-related variance components for facial emotion expressions.* Values are derived from mixed-effects models including group, sex, and age as fixed effects and assessor as a random intercept. The intraclass correlation coefficient (ICC) reflects the proportion of total variance attributable to assessor. Assessor variance represents between-assessor variability, whereas residual variance reflects within-assessor and participant-level variability not explained by the model.

Table S3*. Group differences in raw ratings from the International Affective Picture System (IAPS).* Values represent group means and standard deviations across each emotion and group. Hedges’ g reflects standardized mean differences between groups (CHR - HC), with positive values indicating higher scores in the CHR group and negative values indicating higher scores in the HC group. Confidence intervals represent 95% intervals around Hedges’ g.

Figure S3. *Scree plots for principal component analysis of FaceReader variables in the CHR sample.* Although four components had eigenvalues greater than 1.0, a clear inflection in after the third component, supporting the retention of three components. The dashed horizontal line indicates the Kaiser criterion.

Figure S4. *Scree plots for principal component analysis of IAPS variables in the CHR sample.* Three components exceeded an eigenvalue of 1.0, which supported a three-component solution. The dashed horizontal line indicates the Kaiser criterion.

Table S4. *Pattern matrix from principal component analysis (PCA) of FaceReader emotion variables using Promax rotation.* Values represent standardized pattern loadings, loadings < .40 are removed for clarity. Principal Component 1 was characterized by strong positive loadings for valence, and strong negative loadings for sad and angry expressions (negative affect component). Principal Component 2 showed strong positive loadings for scared and surprised expressions (high arousal component). Principal Component 3 loaded predominantly on happy expression (happy component).

Table S5. *Pattern matrix from principal component analysis (PCA) of International Affective Picture System (IAPS) task variables using Promax rotation.* Values represent standardized pattern loadings; loadings < .40 are removed for clarity. Principal Component 1 reflected general emotional reactivity, characterized by congruent positive and negative emotional responses to pleasant and unpleasant stimuli, respectively. Principal Components 2 and 3 reflected emotional ambivalence, distinguished by opposite directions of the stimulus-rating mismatch (negative emotion to pleasant stimuli for PC2 and positive emotion to unpleasant stimuli for PC3).

Fig S5. *Correlations between facial expression components and negative symptoms in the CHR sample (uncorrected).* (A) Lower scores on the FaceReader High Arousal component were associated with higher NSI-PR EE scores, indicating greater expressivity impairment (p = .027). (B) Higher scores on the FaceReader Happy component were associated with higher NSI-PR MAP scores, indicating greater motivation/pleasure impairment (p = .044).

Fig S6. *Association between IAPS general emotional reactivity and social functioning.* Higher scores on the general emotional reactivity component (reflecting stronger congruent emotional responses to pleasant and unpleasant stimuli) were associated with higher current social functioning (r = .25, p = .028; uncorrected).

Table S6: *Two-step sequential mixed-effects models examining whether FaceReader components explain variance in clinical outcomes over and above emotional reactivity, adjusted for sex and age, with assessor included as a random intercept.* In Step 1, participant sex and age, and a single IAPS emotional reactivity principal component were entered as fixed-effect predictors. In Step 2, one FaceReader-derived component, selected based on the correlation matrix, was added to each model. All models included assessor as a random intercept to account for rater-related variance. Model comparisons reflect the improvement in model fit associated with adding the FaceReader component over and above IAPS emotional reactivity. P values correspond to the χ² test; an asterisk denotes p < .05. After FDR correction, the association between the happy facial expression component and NSI-PR Motivation and Pleasure remained significant (p = .036).
