# Supplementary figures and images for: Computerized assessments of emotional expression and emotional reactivity predict negative symptoms in individuals at clinical high-risk for psychosis
Source: Psychol Med. 2026 Jun 10;56:e184. doi: 10.1017/S0033291726104826 (PMC13280694; doi:10.1017/S0033291726104826)

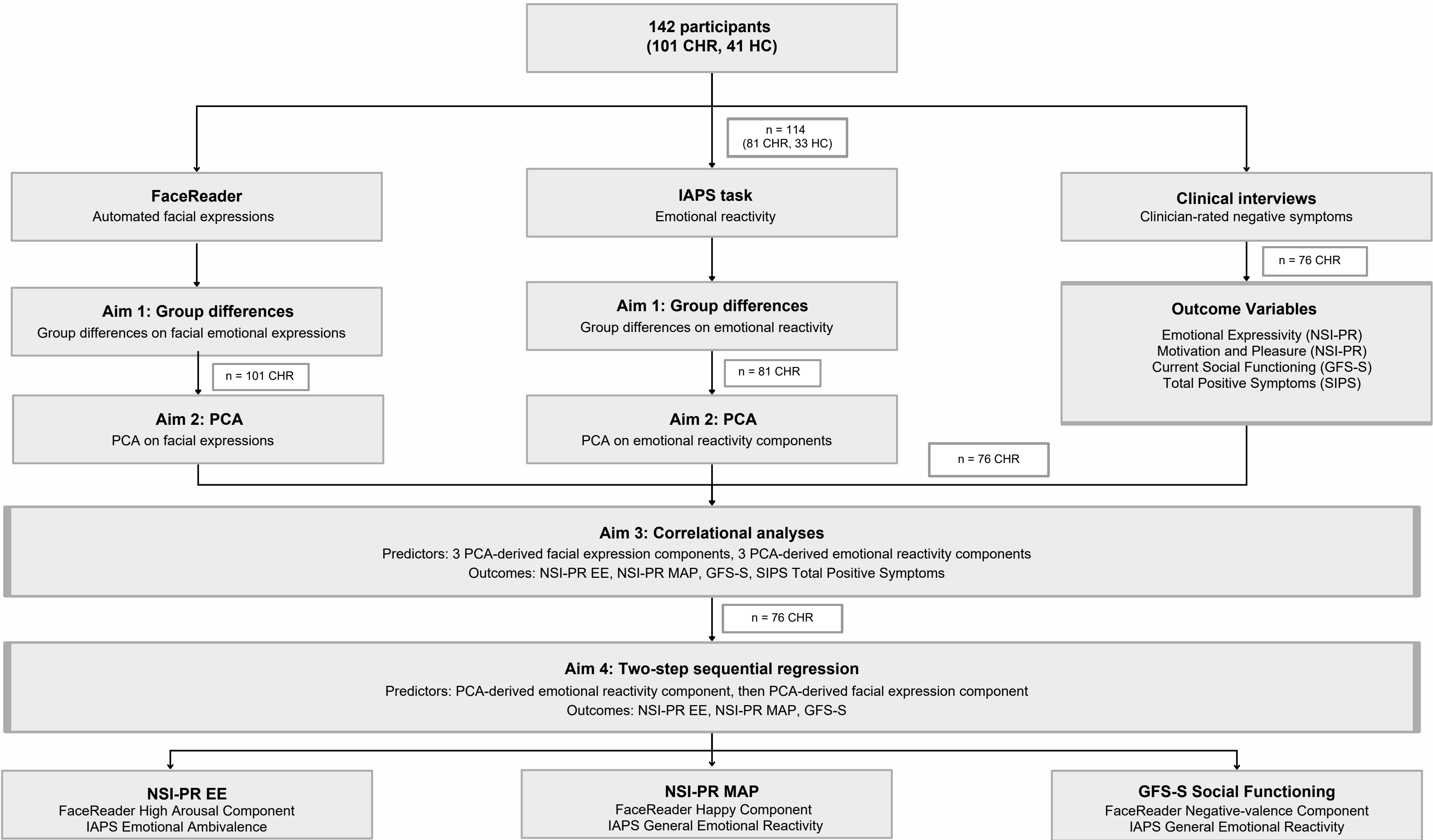

Supplement: Bertrand et al. supplementary material [file S0033291726104826sup001.zip › Supplementary_Figure_S1_BW.pdf]

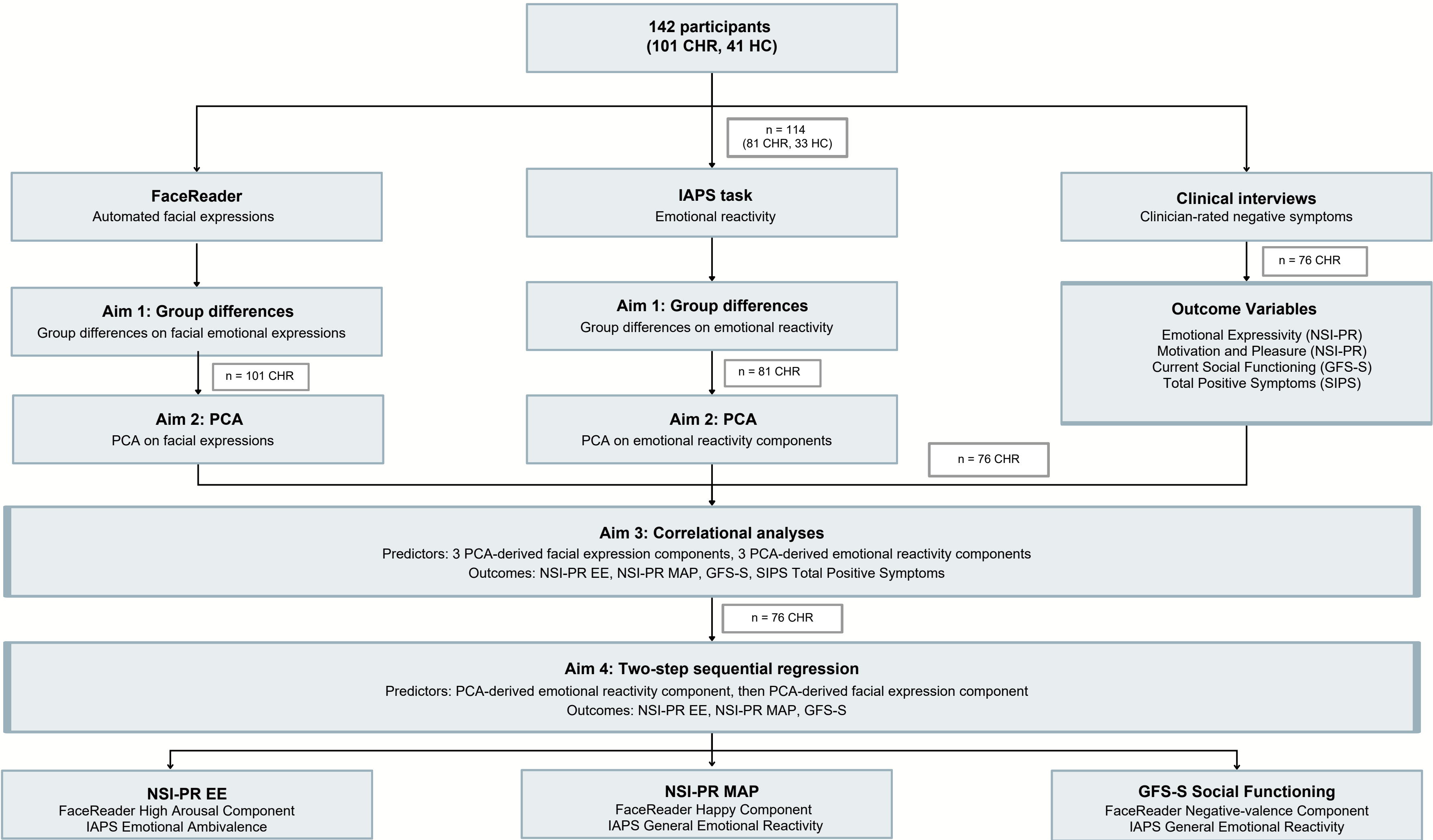

Supplement: Bertrand et al. supplementary material [file S0033291726104826sup001.zip › Supplementary_Figure_S1_COLOR.pdf]

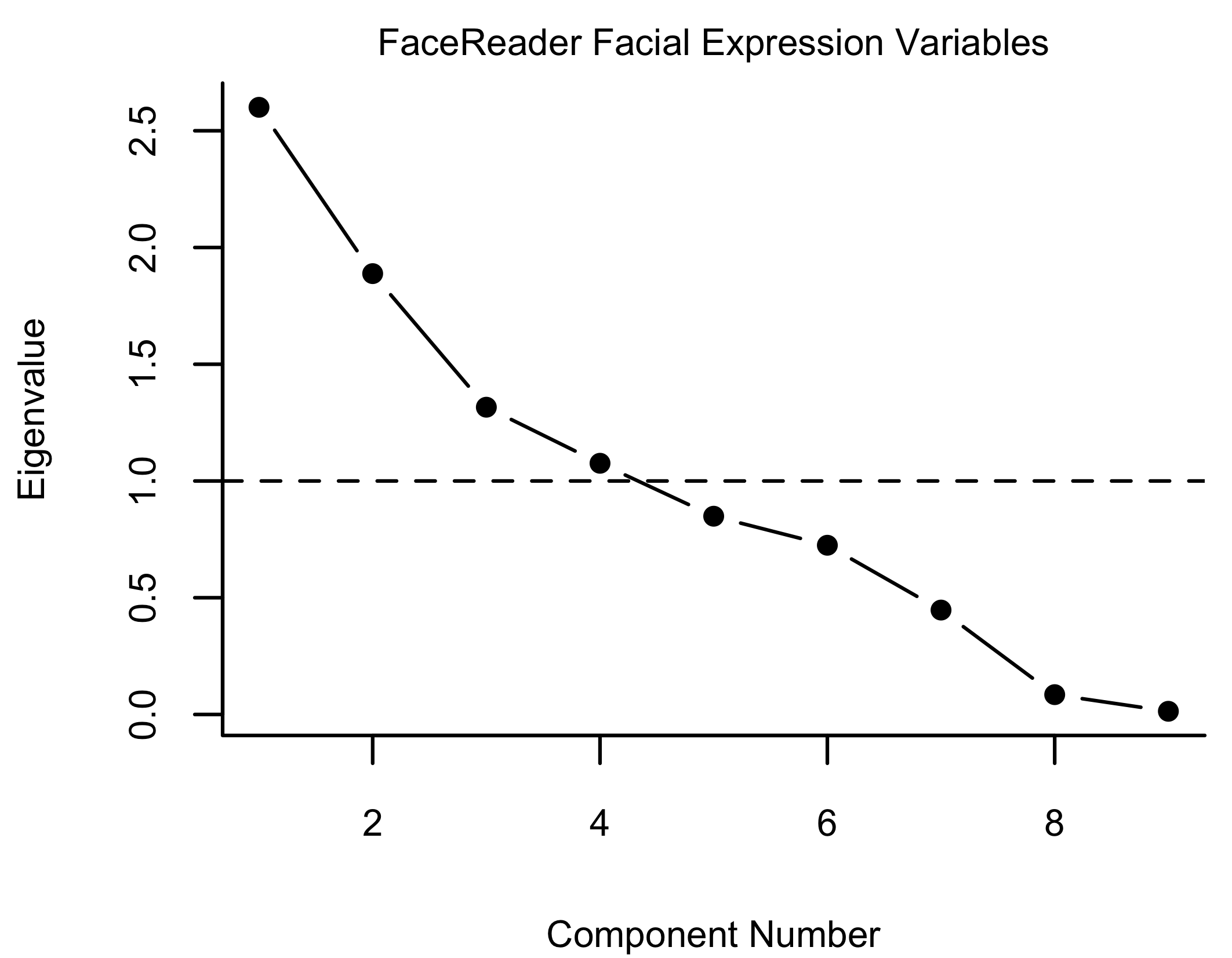

Supplement: Bertrand et al. supplementary material [file S0033291726104826sup001.zip › Supplementary_Figure_S3_BW.tif]

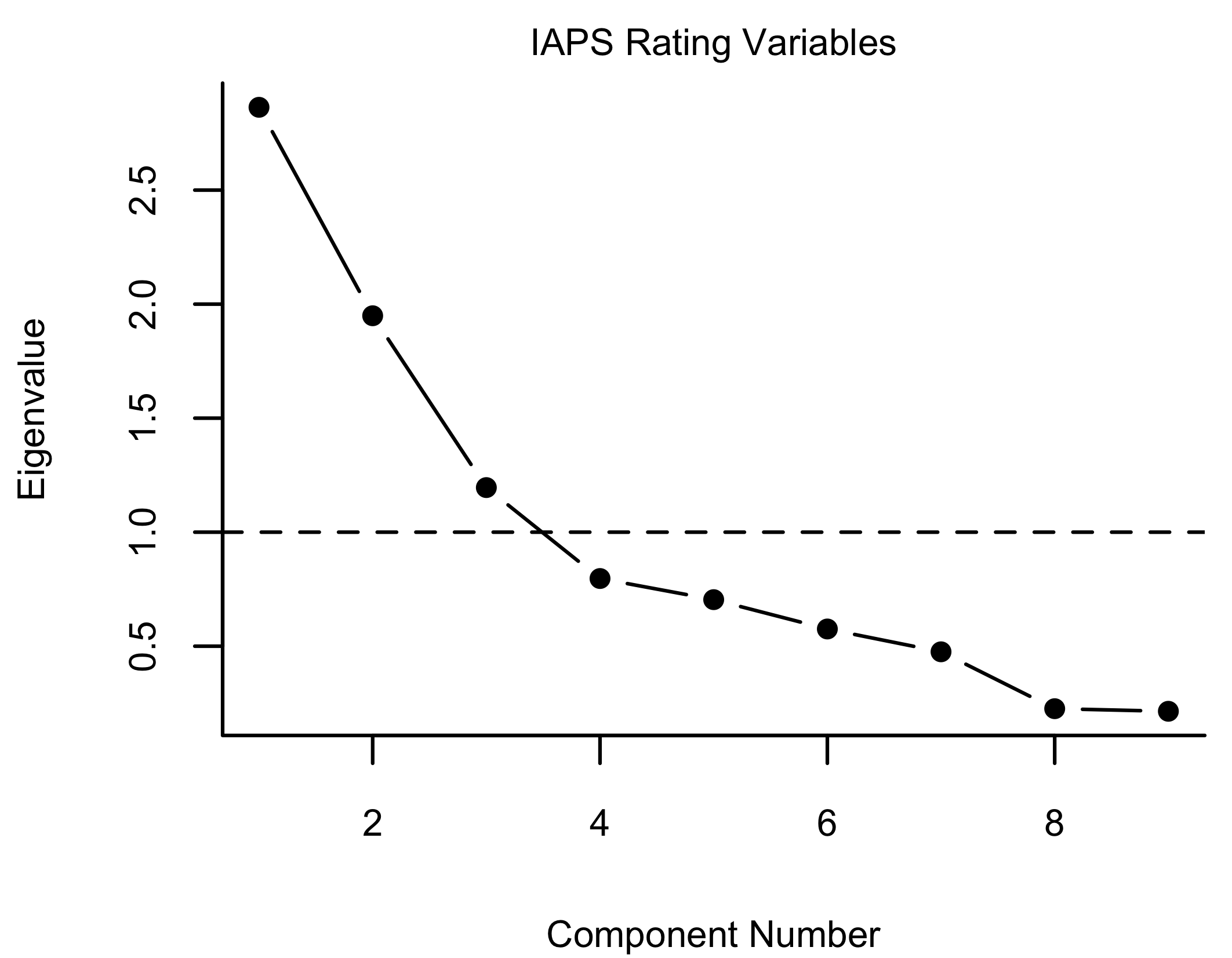

Supplement: Bertrand et al. supplementary material [file S0033291726104826sup001.zip › Supplementary_Figure_S4_BW.tif]

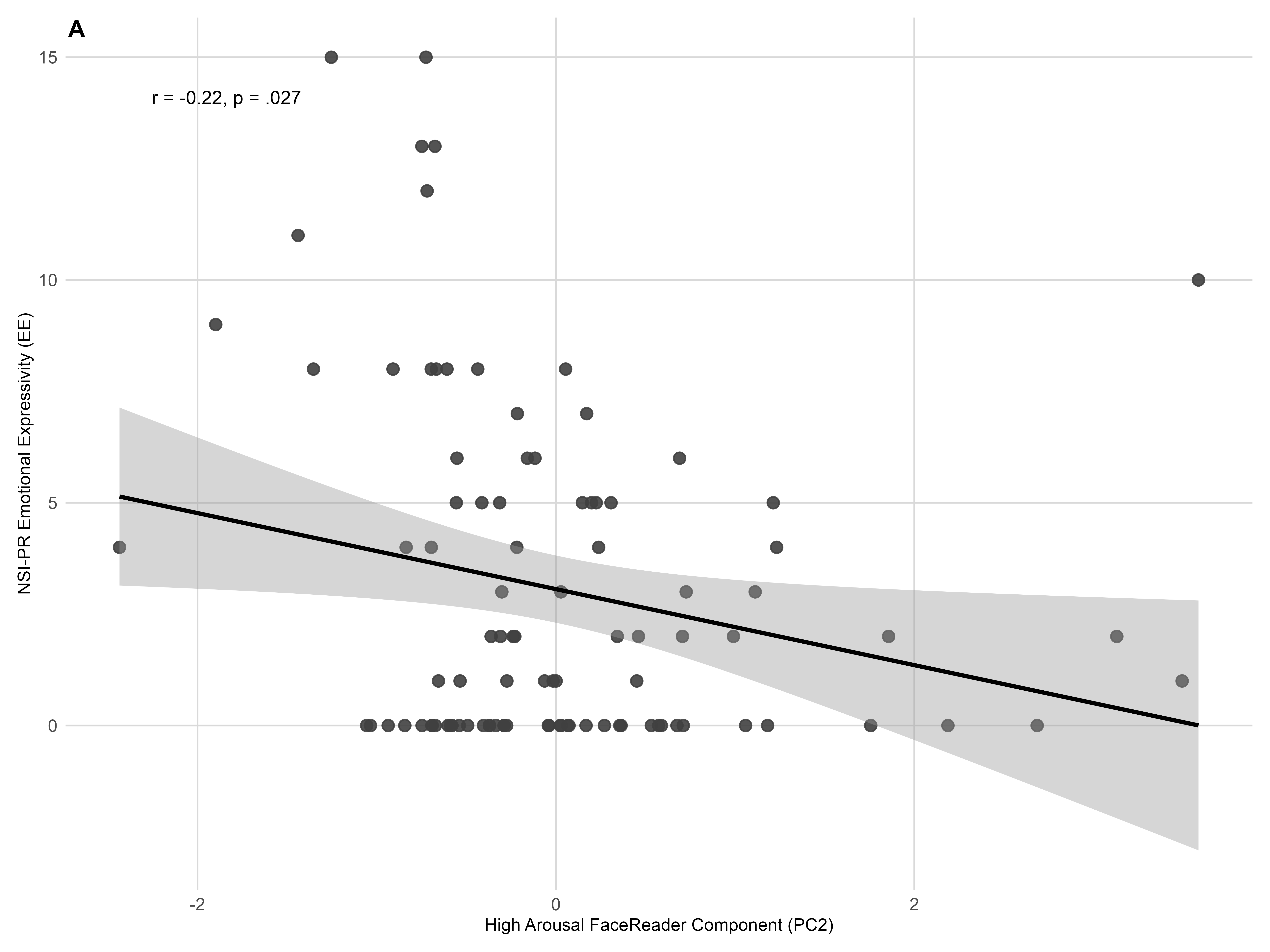

Supplement: Bertrand et al. supplementary material [file S0033291726104826sup001.zip › Supplementary_Figure_S5A_BW.tif]

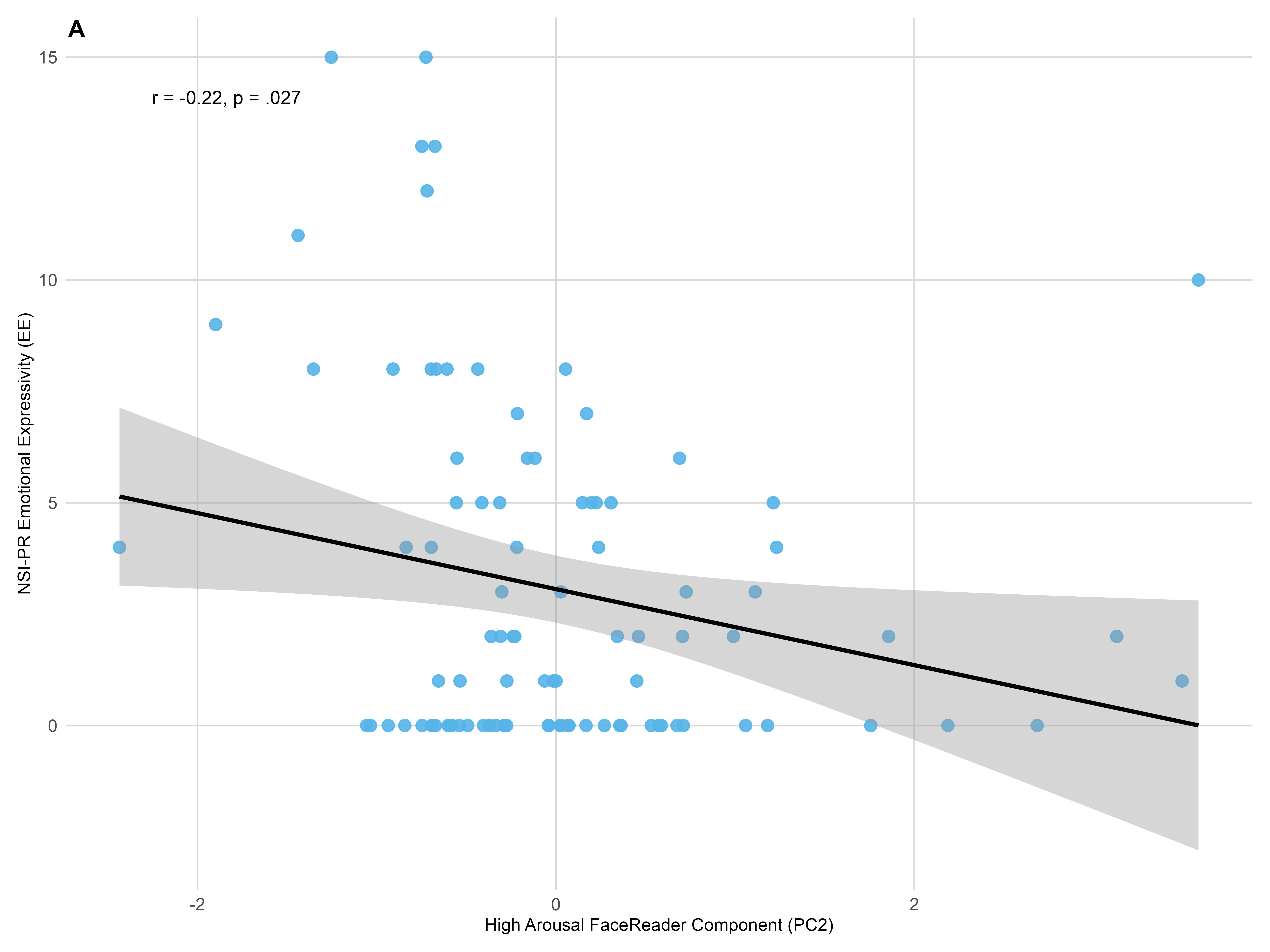

Supplement: Bertrand et al. supplementary material [file S0033291726104826sup001.zip › Supplementary_Figure_S5A_COLOR.tif]

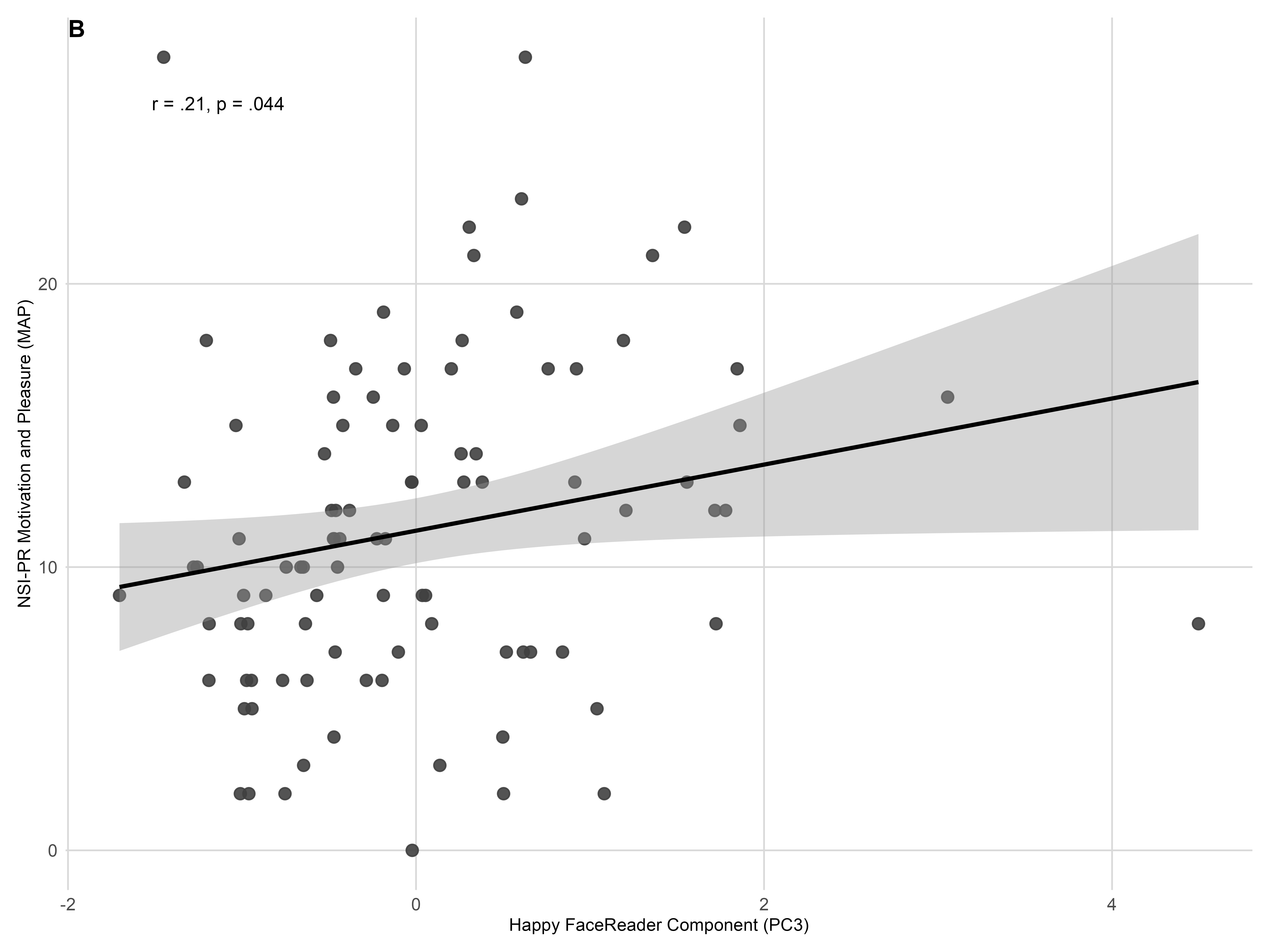

Supplement: Bertrand et al. supplementary material [file S0033291726104826sup001.zip › Supplementary_Figure_S5B_BW.tif]

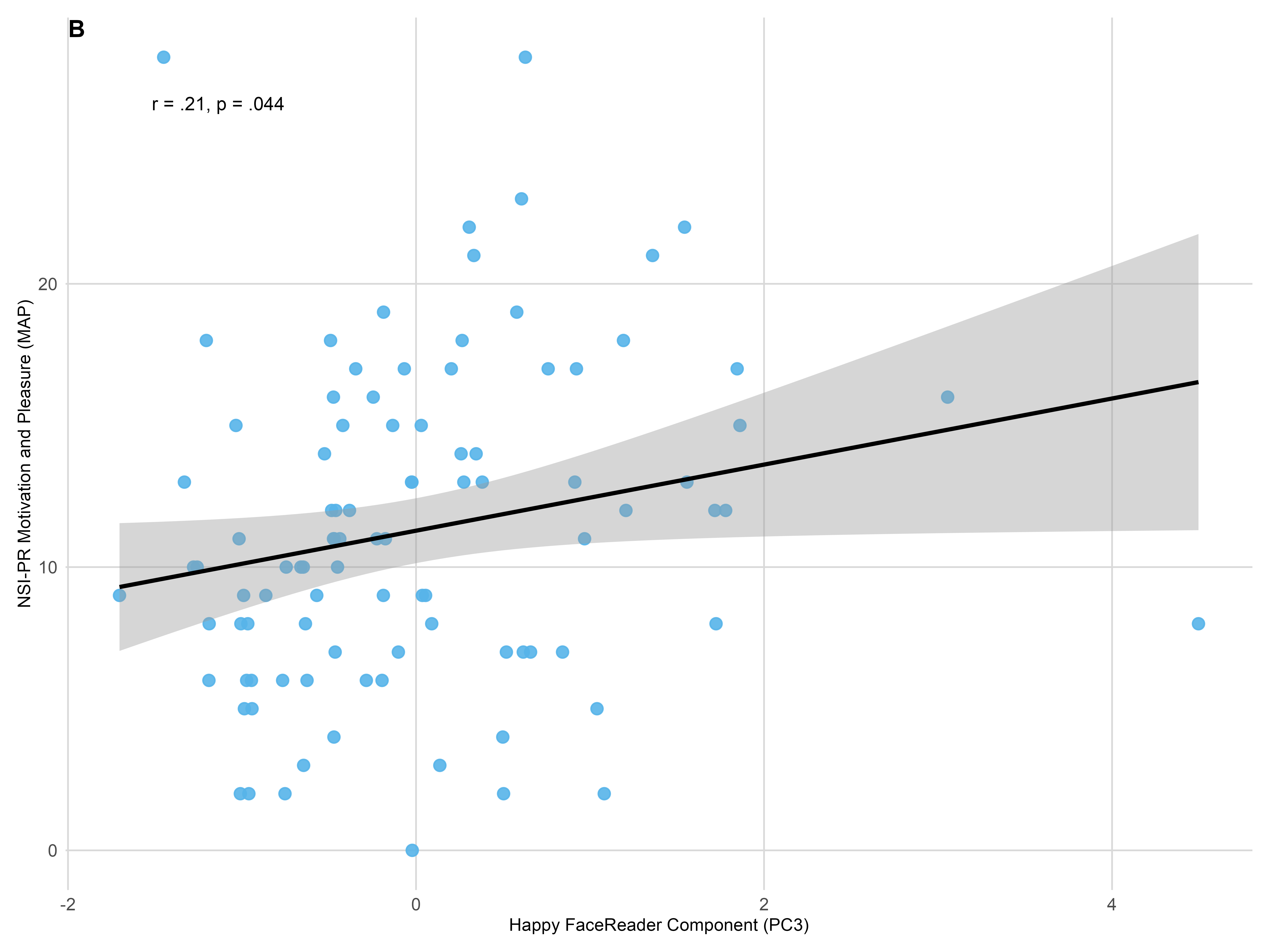

Supplement: Bertrand et al. supplementary material [file S0033291726104826sup001.zip › Supplementary_Figure_S5B_COLOR.tif]

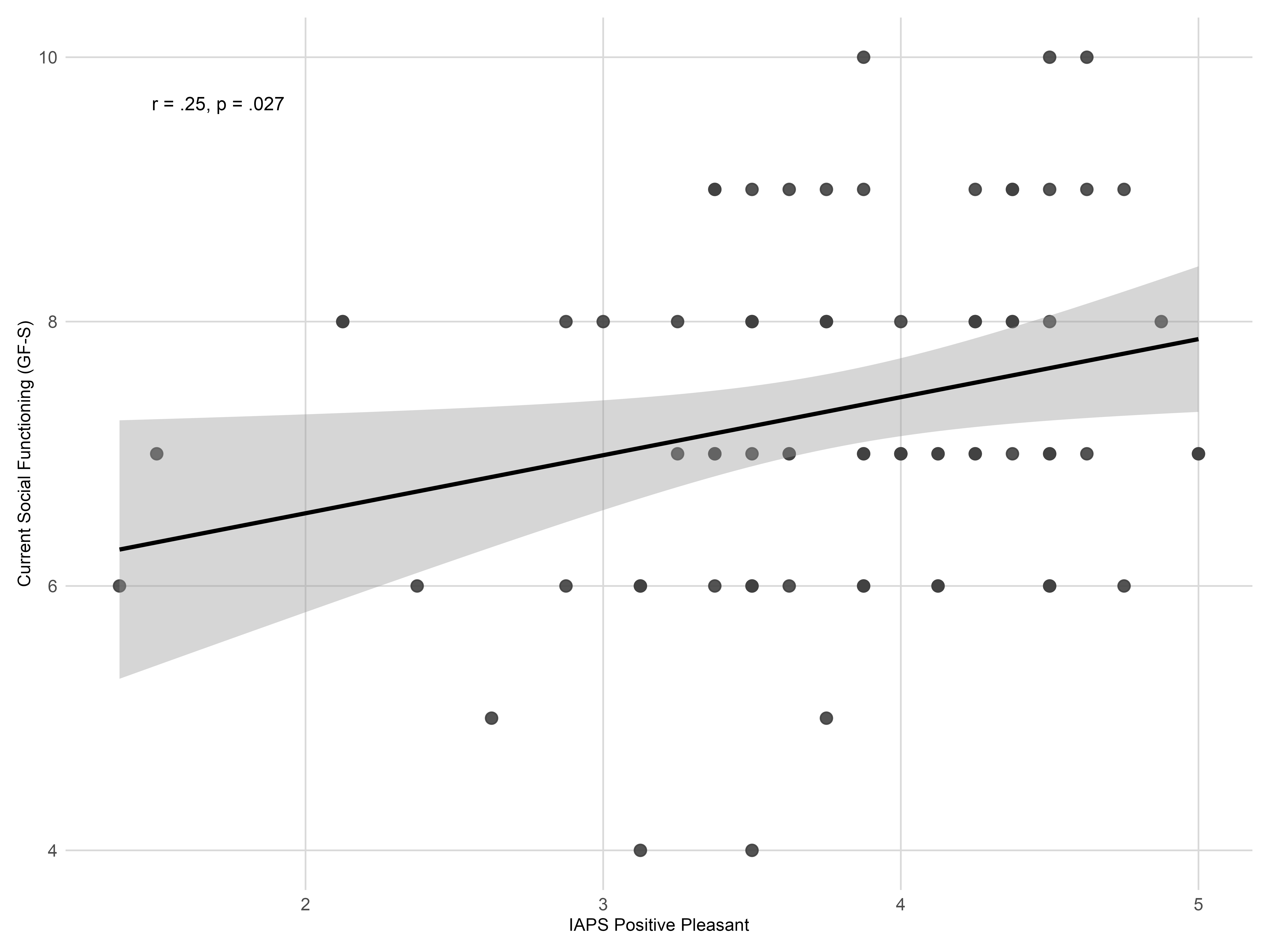

Supplement: Bertrand et al. supplementary material [file S0033291726104826sup001.zip › Supplementary_Figure_S6_BW.tif]

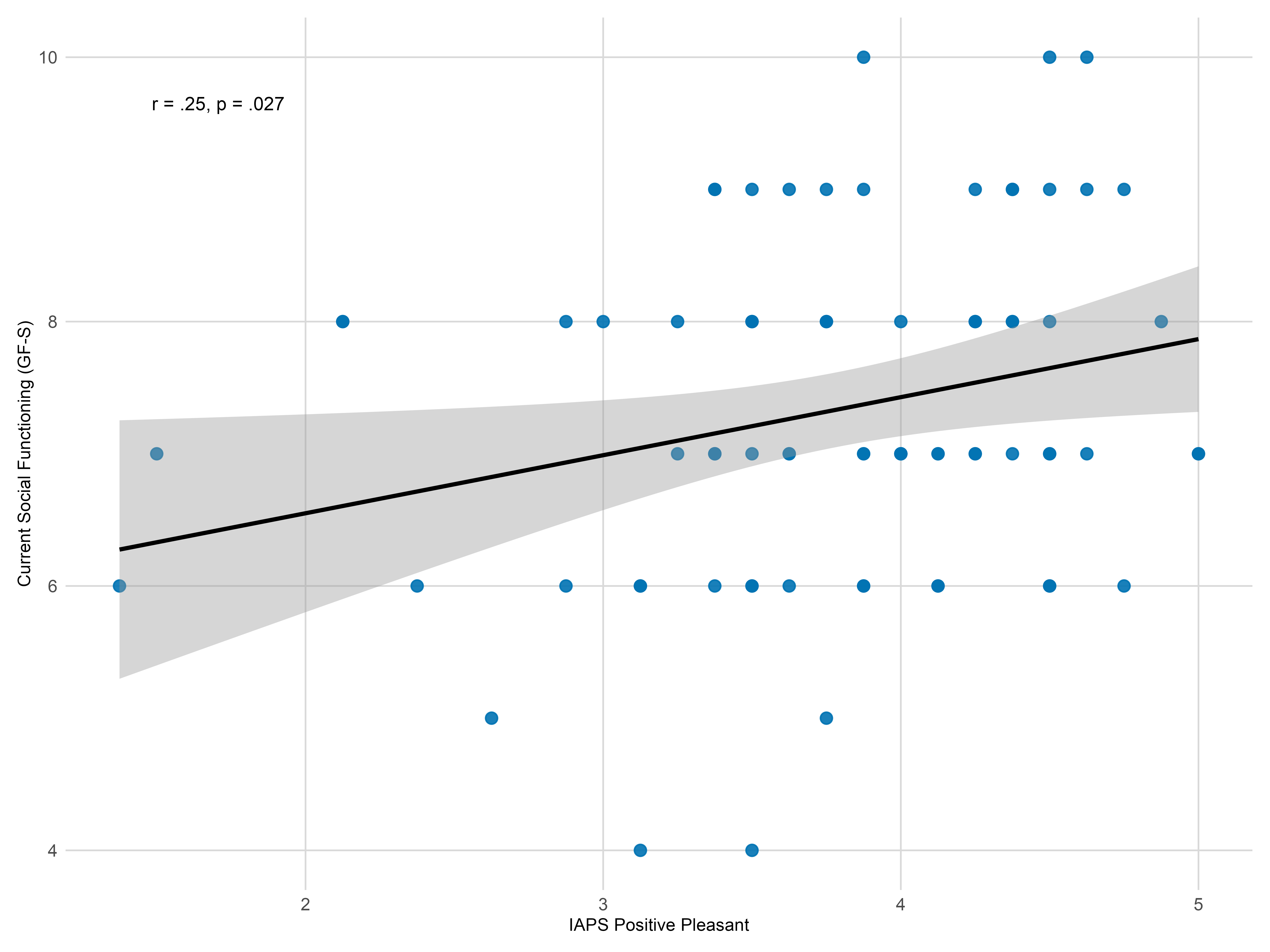

Supplement: Bertrand et al. supplementary material [file S0033291726104826sup001.zip › Supplementary_Figure_S6_COLOR.tif]
